# Volatile Compounds in Pulses: A Review

**DOI:** 10.3390/foods10123140

**Published:** 2021-12-18

**Authors:** Adeline Karolkowski, Elisabeth Guichard, Loïc Briand, Christian Salles

**Affiliations:** 1 CSGA (Centre des Sciences du Goût et de l’Alimentation), AgroSup Dijon, CNRS, INRAE, Université de Bourgogne-Franche Comté, 21000 Dijon, France; adeline.karolkowski@inrae.fr (A.K.); elisabeth.guichard@inrae.fr (E.G.); loic.briand@inrae.fr (L.B.); 2 Ets J. Soufflet, CRIS (Centre de Recherche et Innovation Soufflet), 10400 Nogent-sur-Seine, France

**Keywords:** pulses, volatile compounds, off-flavour, free fatty acid oxidation, amino acid degradation, odour-active compounds

## Abstract

The worldwide demand for pulse-based products is increasing in the face of climate change, but their acceptability is limited due to the presence of off-flavours. Off-notes contribute to negative perceptions of pulses (beany notes). Volatile compounds belong to a large variety of chemical classes. They are mainly produced from the oxidation of unsaturated free fatty acids and the degradation of amino acids during seed development, storage, and transformation (dehulling, milling, and starch or protein production). This review aims to provide an overview highlighting the identification of these molecules in different pulses, their potential origins, and their impact on perceptions. However, data on odour-active compounds in pulses are sparse, as they are limited to those of two studies on peas and lupins. A better knowledge of the volatile compounds involved in the off-notes and their origins should allow for drawing efficient strategies to limit their impact on overall perception for more acceptable healthy food design.

## 1. Introduction

Pulses (*Fabaceae*) are an interesting alternative to animal proteins. They are rich in proteins, unsaturated fatty acids, and bioactive compounds and thus present nutritional and environmental benefits [1,2,3,4,5]. Moreover, they offer functional properties for food applications [2,3]. Many forms are available, such as whole seed, flour, starch, and protein concentrate/isolate [3]. Despite all of the interest in pulses, the presence of off-flavours is in some cases a barrier to their consumption by humans and limits their expansion [4,5,6].

Off-flavours or unpleasant flavours are related to negative organoleptic perceptions. They originate from different volatile molecules responsible for off-notes (unpleasant odours) and from sapid compounds [6]. These tasting compounds activate bitter taste receptors (TAS2Rs) located on the tongue and in the oral cavity. Astringent molecules can precipitate salivary proteins and lead to a loss of lubrification in the mouth [7]. Some saponins, phenolic compounds, alkaloids, peptides, and free amino acids contribute to pulse bitterness whereas phenolic compounds also seem to be involved in astringent sensations [6].

Many volatile compounds have been identified in pulses, and they are mostly responsible for unpleasant odours [6]. These compounds mainly originate from free fatty acids present in the grains and are oxidized into smaller molecules. This phenomenon naturally occurs in the grains but is intensified under stress conditions (water stress or mechanical or herbivore/insect attacks). The production of these compounds contributes to a defence mechanism for these plants and can continue after harvesting [8]. Small compounds, such as aromatic hydrocarbons, aldehydes, alkanes, alkenes, alcohols, ketones, acids, esters, pyrazines, terpenes, furans, and lactones, have been identified in pulses [6]. Even though each of these molecules has a specific odour, the perception of an aroma is often due to a mixture of different notes from several molecules [9]. Off-notes in pulses are described as beany, green, pea-like, earthy, hay-like, fatty, pungent, and metallic [6].

This review is focused on dried legume seeds, also called pulses, such as black beans, pinto beans, and dark red kidney beans, which are known as common beans (*Phaseolus vulgaris*); peas (*Pisum sativum*); broad beans (*Vicia faba*), also called faba beans; and chickpeas (*Cicer arietinum*). Even if soybean (*Glycine max*) is considered a pulse, it will not be considered in the present review because of its high oil concentration. The first part of the manuscript presents the main origins of volatiles identified in pulses, including the oxidation of oleic, linoleic, and linolenic acids (enzymatic and nonenzymatic pathways); the degradation of free amino acids by multiple pathways; and the degradation of carotenoids. The second part addresses the different methods used to extract volatile compounds from pulses. The most common methods are Solvent-Assisted Flavour Evaporation (SAFE) and HeadSpace Solid-Phase MicroExtraction (HS-SPME). The third part inventories the volatile compounds identified in different pulses, arranged by class of molecules, and their origins are given. Different parameters, such as the type of pulses, cultivar, year and location of culture, and transformation process (conditions of storage, dehulling, grinding, and starch and protein production) were selected to highlight their role in the production of volatiles. The last part is devoted to odour-active compounds in pea and lupin flours. For each compound, odour descriptors and threshold detection are given.

## 2. Origins of Volatile Compounds in Pulses

The generation of volatiles takes place during seed development and continues after harvest during storage and seed transformation. It begins during the physiological processes of plants, but it can be more intense under stressful conditions, such as mechanical wounding/herbivores, pathogen infection, or temperature/water stresses [8]. Therefore, this production of volatiles is a defence mechanism for plants [10]. Seeds that have been harvested in a timely manner should present lower concentrations of volatiles [11]. Three main sources of volatile production are highlighted: the oxidation of unsaturated free fatty acids, amino acid degradation, and carotenoid degradation.

### 2.1. Oxidation of Unsaturated Free Fatty Acids

**Figure 1:**
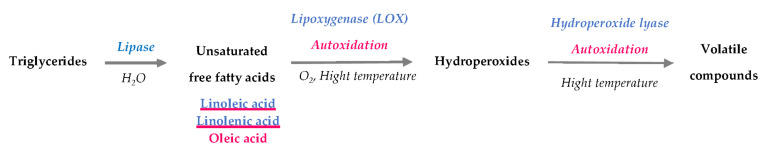
Synthesis of volatile compounds from pulse triglycerides (adapted with permission from [13,14,15]; Copyright (2021) Elsevier). (enzymatic pathways in blue and nonenzymatic pathways in pink).

Different classes of volatiles, such as aromatic hydrocarbons, aldehydes, alcohols, alkanes, ketones (furans) and esters, are mostly derived from enzymatic or nonenzymatic oxidation (autoxidation) of free fatty acids. Although pulses have a low fat content (0.8–7% of seed weight), this mechanism is dominant and strongly contributes to unpleasant odours, such as herbal, green, pea, beany, mould, and rancid notes [12]. The synthesis of these compounds follows three phases (Figure 1).

#### 2.1.1. Triglyceride Hydrolysis

**Figure 2:**
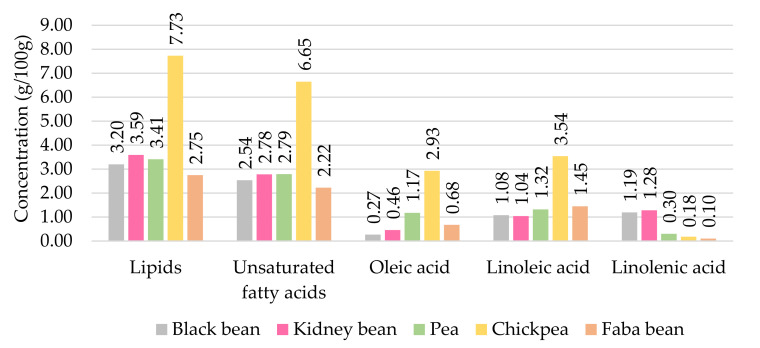
Amount of lipids and fatty acids in pulses (g/100 g) (adapted from [17]).

Pulses contain a low amount of free fatty acids, as fat is mainly composed of triglycerides and phospholipids. The composition and concentration of lipids depend on the variety, origin, location, type of soil where the seed is cultivated, climate, and seasonal and environmental conditions. Moreover, among the free fatty acids precursors of volatile compounds, mature seeds contain a higher amount of oleic acid, whereas linoleic acid is more abundant than oleic acid in mature seeds [16]. Chickpeas contain two to three times more lipids than the other pulses. (Figure 2). However, black beans and kidney beans contain more linolenic acid than oleic acid compared to the proportion in peas, chickpeas, and faba beans. The diversity of lipid profiles in pulses contributes to the large variety of volatile compounds.

Lipase (triacylglycerol acylhydrolase, EC 3.1.1.3) hydrolyses triglycerides and releases the corresponding free fatty acids, with linoleic acid being the predominant fatty acid produced. This enzyme has a stronger activity towards short-chain fatty acid glycerides [13]. During storage, the amount of free fatty acids increases in faba beans [18]. In addition, lipase also hydrolyses phospholipids, but the amount of free fatty acids released represents 1 to 3% versus 10 to 20% from triglycerides in oat varieties [19].

#### 2.1.2. Oxidation of Free Fatty Acids

Free fatty acids, such as linoleic and linolenic acids, are the substrate of lipoxygenase (LOX), a nonheme iron dioxygenase that catalyses the addition of O_2_ in polyunsaturated fatty acids with a *cis, cis*-1,4-pentadiene moiety [20].

The oxidation of free fatty acids follows three stages. First, unsaturated fatty acids (RH) lose hydrogen in the presence of a pro-oxidant initiator, such as LOX, that promotes the formation of an alkyl radical (R^•^). The initiation step is also induced by nonenzymatic factors, such as high temperatures, light, and metal ions. Moreover, tissue disruption or frost activates enzymatic oxidation of legumes in the field or during storage [11]. During autoxidation, oleic, linoleic, and linolenic acids are also oxidized into hydroperoxides [14]. Then, during the propagation step, O_2_ reacts with the alkyl radical (R^•^) to form a peroxy radical (ROO^•^) that is reduced to an unstable hydroperoxide (ROOH) by oxidizing another fatty acid (RH). A new alkyl radical (R^•^) is formed. Thus, this radical could react with a new molecule of O_2_ and lead to new hydroperoxides. Finally, peroxy radicals (ROO^•^) react together to produce non-radical products during the termination step [21].

- *Initiation:*  RH → R^•^ + H^•^
- *Propagation:* R^•^ + O_2_ → ROO^•^
        ROO^•^ + RH → ROOH + R^•^
- *Termination:* ROO^•^ + ROO^•^ → Non-radical products

Different LOX isoenzymes have been identified in pulses. Isoenzymes differ in their optimal pH and the hydroperoxides produced. Yoon and Klein (1979) isolated four pea LOX isoenzymes [22]. The major enzymes PL-I and PL-II have an optimum pH between five and seven but are inactivated above eight. They are characterized by different pH profiles, substrate specificities, and abilities to produce hydroperoxides. In chickpeas, two isoenzymes are present, namely CL-1 and CL-2. CL-1 has an optimal pH of 6.0 and an apparent Km for linoleic acid of 192 µM, whereas CL-2 has a pH of 5.5 and an apparent Km of 51 µM. CL-1 is more thermostable than CL-2, and its specific activity decreases with enzyme concentration [23]. Moreover, in broad beans (faba beans), two isoenzymes, BBL-1 and BBL-2, were purified by Clemente et al. (2000) [24]. These isoenzymes present different optimal pH values of 5.6 for BBL-1 and 5.8 for BBL-2. Their Km values for linoleic acid are 2.3 mM and 0.25 mM. These enzymes also produce different proportions of hydroperoxides: BBL-1 releases 62.1% 9-hydroperoxides and 37.9% 13-hydroperoxides, whereas BBL-2 produces 90.3% 13-hydroperoxides and 9.7% hydroperoxides.

#### 2.1.3. Degradation of Hydroperoxides and Formation of Volatile Compounds (Secondary Products)

The degradation of hydroperoxides involves a complex set of reactions that produces many volatile and non-volatile compounds that contribute to the global flavour. Hydroperoxides are unstable molecules that are decomposed into small molecules under the effect of heat or metal ions (autoxidation) or in the presence of hydroperoxide lyase.

The diversity of hydroperoxides promotes a variety of different volatile compounds. Oxidation of oleic acid leads to the formation of 8-, 9-, 10-, and 11-hydroperoxides, whereas oxidation of linoleic acid leads to two stereoisomers of 9- and two stereoisomers of 13-hydroperoxides. The oxidation of linolenic acid produces 9-, 12-, 13-, and 16-hydroperoxides. Furthermore, 9-hydroperoxide mainly leads to 2,4-decadienal, while 13-hydroperoxides produce hexanal. Other aldehydes, acids, ketones, or lactones originate from hydroperoxide degradation [14]. Subsequently, aldehydes are converted into alcohol by alcohol dehydrogenase [25].

### 2.2. Degradation of Free Amino Acids

The degradation of amino acids has been shown to be the second source of volatile compound production in pulses. Several origins exist, such as biodegradation in seeds, degradation by microorganisms, and Maillard reactions.

#### 2.2.1. Biodegradation

Amino acids are the source of a myriad of volatile compounds, such as branched-chain compounds, benzene aldehydes, alcohols, acids, esters, nitrogen compounds (amines, pyrazines and pyridines), and sulphur compounds. Their origins are the breakdown of proteins by proteases and the hydrolysis of peptides. Spinnler (2011) studying the amino acid degradation pathways in microorganisms, assumed that these pathways could also occur in plants [26]. In addition, certain enzymes involved in this degradation are present in tomatoes and bananas [25].

The Ehrlich−Neubauer pathway is the most commonly used pathway by microorganisms. The amino acids are converted into an α-ketoacid by a transaminase. This α-ketoacid is then decarboxylated into aldehyde, alcohol, and acid (the latter two have one less carbon than the original amino acid). Alanine, phenylalanine, valine, methionine, isoleucine, and leucine are precursors of volatile compounds (Table 1). Pulses are deficient in methionine compared to cereals [27]. This could explain the absence of alcohols and acids derived from these amino acids in the studied pulses.

#### 2.2.2. Degradation by Microorganisms

The presence of unwanted microorganisms in pulses promotes the formation of volatile compounds from amino acids. In blue lupins (*Lupinus angustifolius*), acids are produced from the metabolism of microorganisms present in the seed coat. The concentration of carboxylic acids increases considerably during storage [28]. The production of acetic acid and 2- and 3-methylbutanoic acids by fermentation is also demonstrated in wheat flour [29]. The metabolic pathways used would be similar to those described by Spinnler (2011) [26]. Moreover, unwanted fermentation by *Pseudomonas perolans* and *taetrolens* leads to the formation of 3-isopropyl-2-methoxypyrazines [30].

#### 2.2.3. Maillard Reactions

One method of amino acid degradation is Maillard reactions in the presence of sugars. These nonenzymatic browning reactions lead to the formation of volatile compounds, such as pyrazines, furanones, pyranones, or thiazoles [31,32]. These reactions occur during long storage or heat treatments in different pulses (chickpeas, faba beans, beans, lentils, peas) [33].

The formation of pyrazines occurs at different steps of the Maillard reactions. First, methoxypyrazines are secondary products of plant metabolism from amino acids and 1,2-dicarbonyl [34]. Jakobsen et al. (1998) identified different 3-alky-2-methoxypyrazines in raw legumes (immature seeds) [35]. 3-Isopropyl-, 3-sec-butyl-, and 3-isobutyl-2-methoxypyrazines derive from valine, isoleucine, and leucine, respectively [25]. Despite their low abundance in legumes, these compounds mainly contribute to aroma due to their very low odour detection threshold (detection threshold in water: 0.001–0.002 µg/L) [34,35]. Second, new pyrazines are formed during the Strecker reaction at temperatures greater than 100 °C [31]. Third, their synthesis is possible at ambient temperature from acetoin (3-hydroxy-2-butanone) and ammonia [36]. Thus, this reaction could occur during prolonged storage. Acetoin was identified in dehulled low- and high-tannin faba beans [37]. Moreover, caramelization also produces volatile and coloured compounds from only sugars in dry processes at high temperatures (100–150 °C). This reaction occurs during the production of faba bean protein concentrates [38].

### 2.3. Degradation of Carotenoids

Terpenes can be derived from the degradation of carotenoids. Carotenoids are oxidized by LOX 2 at neutral pH and produce these volatile compounds [39]. This origin is highly disputed. Indeed, due to the low concentrations found in plants, these molecules would be absorbed by plant roots at the soil level during their cultivation and then accumulate in the seeds [25].

## 3. Extraction, Separation, Identification, and Semi-Quantification Methods

The characteristics of the studied pulses (cultivar and year and location of cultivation), conditions of storage, transformation, and volatile compound analysis are described in Table 2 [11,37,40,41,42,43,44,45]. Different classical methods are used to extract volatile compounds, which are briefly described here. By using HS-SPME (HeadSpace Solid-Phase MicroExtraction), the volatile compounds in the vapour phase are first adsorbed on a fibre and desorbed in the GC (Gas Chromatography) injector to be separated and identified. This method is robust, rapid, simple to use, and solvent-free but allows only semi-quantification due to competition between analytes on the fibre [46,47,48]. SAFE (Solvent-Assisted Flavour Evaporation) combines vacuum distillation and solvent extraction [49]. This method allows quantification by a standard but requires a long extraction time and requires the use of organic solvents to extract volatiles [48]. The differences highlighted in these two methods could also explain some differences between pulse volatile compounds.

## 4. Identification and Quantification of Volatile Compounds in Pulses

In Table 3, Table 4, Table 5, Table 6, Table 7, Table 8, Table 9, Table 10, Table 11, Table 12, Table 13 and Table 14, each table describes a chemical class of volatile compounds: aromatic hydrocarbons, aldehydes, alkanes/alkenes, alcohols, ketones, acids, esters (without lactones), pyrazines, terpenes, furans, lactones, and other volatile compounds. For each pulse, one column corresponds to the minimum and the maximum (min–max) of a set of different cultivars, harvest years, locations and conditions of storage, or seed transformations (dehulling and production of protein concentrates or isolates) (see Table 2 for more details).

For more homogeneity, the amounts of volatile molecules are given as a relative percentage of the total amount of volatile compounds (calculated from the peak areas (GC-MS) or concentration). Unfortunately, most of the quantification is given as peak area or relative percentage instead of real concentration [11,37,40,41,43,44,45]. To obtain comparable data, quantification is expressed as a percentage in all pulses in this review. Moreover, it is important to note that in some studies, the total percentage of peak area of concentration is not equal to 100%. For the three cultivars “Whole Pea” [11], the total percentage is approximately equal to 50% and approximately 75–85% for the two cultivars “Chickpea” [43].

### 4.1. Aromatic Hydrocarbons

Aromatic hydrocarbons (Table 3) are present at low percentages for all pulses except styrene (sweet, balsamic, and almost floral odour [50]). Pea proteins (concentrate and isolate) do not contain aromatic hydrocarbons, and chickpeas only have styrene. Common beans (black beans, pinto beans, and dark red kidney beans) and faba beans (tannins and storage) have a higher percentage of aromatic hydrocarbons than peas (whole and dehulled) and faba beans (location). The main origin of these compounds is free fatty acid oxidation. Common beans (black beans, pinto beans, and dark red kidney beans) present a high percentage of styrene. Oomah et al. (2007) showed that contamination or extraction problems cannot explain this phenomenon; however, a similar amount was quantified in “organic” beans samples, suggesting the intrinsic origin of this compound [40]. These beans were stored at 23 °C with a relative humidity of 15–20% before analysis (no information on storage time is given). It can be supposed that these storage conditions are favourable to the enzymatic oxidation of free fatty acids and promote the synthesis of styrene and other identified aromatic hydrocarbons [45,51].

As the extraction, separation, and identification methods used are identical for common beans (black beans, pinto beans, and dark red kidney beans) and faba beans (location), the data can be compared [40,41]. Faba beans contain less styrene, cumene, and p-xylene than common beans but more ethylbenzene. The percentages of toluene are similar. Moreover, 1,2,3-trimethylbenzene, 1,3,5-trimethylbenzene, and propylbenzene are absent in faba beans.

### 4.2. Aldehydes

Aldehydes (Table 4) constitute the main chemical class identified in pulses except for common beans (black beans, pinto beans, and dark red kidney beans) that contain a high percentage of styrene (aromatic hydrocarbon). Hexanal (fatty, green, and grassy odour [50]) is predominant, and its percentage represents half of the total aldehyde percentage for all pulses. Free fatty acid oxidation is responsible for 15 compounds and the amino acid degradation for six compounds (the origins of nine compounds are not identified). Whole peas and dehulled peas contain fewer aldehydes than other pulses, whereas pea proteins (concentrate and isolate) and faba beans have a high percentage of aldehydes.

For black beans, the Onyx cultivar has more hexanal than the AC Harblack cultivar. This difference could be explained by their different fatty acid compositions, especially for linoleate, which is responsible for hexanal synthesis [40]. Aliphatic unsaturated aldehydes, such as (E)-2-hexenal, (E)-2-heptenal, (E,E)-2,4-heptadienal, (E)-2-octenal, and (E)-2-nonenal, could originate from physical damage caused by tissue disruption or frost damage that could induce oxidation of free fatty acids [11,40].

For whole peas, a difference between the three crop years was highlighted: the 2005 crop had the greatest percentage of aldehydes. (E)-2-Octenal (green-leafy odour [50]) was the predominant aldehyde in 2005 and 2007, whereas hexanal was the major aldehyde in the 2006 cultivar [11]. These results suggest that environmental stress conditions associated with the culture, harvesting time, and uncontrolled storage conditions could be the causes of these differences. Dehulled peas contain the lowest percentage of aldehydes [42], whereas other studies show an increasing concentration of aldehydes after dehulling [11,37]. Dehulled peas were stored at negative temperature, whereas the other pulses were stored at 4 and 18 °C. Thus, these higher temperatures enable lipoxygenase oxidation and promote volatile production, which could explain the different observations.

Concerning pea proteins, there are some differences between the concentrate (dry process) and isolate (wet process). In particular, concentrates do not contain benzaldehyde, 4-ethylbenzaldehyde, phenylacetaldehyde, vanillin, pentanal, furfural, (E)-2-hexenal, and (E)-2-octenal, whereas 3-methylthiopropanal, 3-methylbutanal, (E)-2-heptenal, octanal, and decanal are not identified in isolates [42,44]. Both pea proteins contain 2-methylbutanal and nonanal. Hexanal represents approximatively 50% of aldehydes in concentrates and more than 65% in isolates. Moreover, aldehydes from amino acids are mainly present in pea proteins. These differences could also be explained by the composition of lipids and proteins: concentrates and isolates contain 55.5 and 77.1% proteins, respectively, and 2.9 and 8.6% lipids [42,44]. Isolates are more exposed to free fatty acid oxidation and degradation of amino acids due their higher concentration of lipids and proteins than those in concentrates.

Concerning the two chickpea cultivars, a few differences were observed between the two cultivars in their percentages of octanal, nonanal and (Z)-2-decenal. (E,E)-2,4-Nonadienal is absent from the Desi cultivar [43].

For faba beans, low-tannin cultivars contain the smallest percentage of aldehydes compared to high-tannin cultivars [37]. The differences in the percentages mainly include hexanal and nonanal. Concerning the faba bean field location, this effect has been demonstrated for pentanal, (E)-2-heptenal, and nonanal percentages. Faba beans from Barrhead present a higher percentage of these volatiles than Namao faba beans [41]. Although the cultivation conditions were not detailed, this could be an explanation for these differences. Moreover, the storage conditions can promote the formation of volatiles in pulses. Faba beans after 60 days of storage at room temperature have a percentage of pea area of 80% versus 30% for refrigerated faba beans and 10% for frozen grains. Lower temperatures limit or block enzymatic reactions [45].

### 4.3. Alkanes and Alkenes

Alkanes (Table 5) are present in pulses except for dehulled peas and chickpeas, whose relative percentages are 1.27% and 0.31–0.57%, respectively [42,43]. Alkanes are not identified in pea protein concentrates (dry process) [44]. The origin of volatiles is mostly free fatty acid breakdown [40]. In common beans (black beans, pinto beans, and dark red kidney beans), high relative percentages are identified in AC Pintoba and Maverick cultivars for pinto beans and CDC Rio and Onyx cultivars for black beans [40]. For whole peas, the 2006 cultivar contains 1.2 times more alkanes than 2005 and 2 times more alkanes than the 2007 cultivars [11]. Regarding concentration, pea protein isolates (wet process) contain approximatively 20 times more alkanes than dehulled peas. High concentrations of tetradecane, pentadecane, and hexadecane were identified in pea isolates. No lipoxygenase activity was identified in pea protein isolates in contrast to dehulled peas [42], and it seems that autoxidation is promoted during protein production due to an elevation of temperature (40 °C). There is no difference in the relative percentage between low- and high-tannin faba beans [37] or between the two locations of faba beans [41]. However, the relative percentages of alkanes decrease after 60 days of storage for each temperature (ambient, refrigerated, and frozen) [45]. As the results are expressed as a relative percentage of peak area instead of concentration, the relative decrease in the percentage of alkanes could be explained by a high increase in the percentage of aldehydes rather than a real increase in the concentration of alkanes.

Concerning alkenes, dehulled peas contain the highest relative percentage due to the presence of 1-tetradecene [42], while this class is not detected in other pulses or only represents a very small percentage. The SAFE method used only by Murat et al. (2013) allows the extraction of compounds with a higher molecular weight than the SPME method [42].

### 4.4. Alcohols

Alcohol (Table 6) is the class with the highest number of volatiles that reflects great diversity. Free fatty acid breakdown is the major origin identified in the production of volatiles, but the degradation of amino acids also leads to the formation of alcohols. Hexanol (herbaceous, woody, and green odour [50]) and octanol (waxy and cheesy fatty aroma [50]) are identified in all pulses except chickpeas.

For common beans, AC Harblack (black beans), ROG 802, and Redhawk cultivars for dark red kidney beans have relative percentages of approximatively 3.6–5.4%, while the other cultivars present percentages between 15.4 and 19.3%. Pentanol, 3-methylbutanol (penetrating green aroma [40]), 2-ethylhexanol (citrus, fresh, and floral odour [42]), octanol, nonanol, benzyl alcohol, and phenol are not identified in any of the samples.

Whole peas contain two alcohols, and their relative percentage is very low. Dehulled peas and pea protein isolates have the same concentration of alcohols (data not shown) [42]. Dehulled peas are richer in 3-methyl-3-pentanol, 1-penten-3-ol (penetrating grassy ethereal odour [40]), (Z)-3-hexen-1-ol (grassy-green odour [50]), and undecanol (floral, citrus-like odour [50]), while pea protein isolates contain a higher percentage of 2-butoxyethanol, pentanol, octanol, 1-octen-3-ol (mushroom odour [42]), and (E)-2-octen-1-ol. In comparison with isolates, pea protein concentrates (dry process) present a higher relative percentage of alcohols, including pentanol, 1-penten-3-ol, 2-penten-1-ol, hexanal, 2-ethylhexanol, heptanol, octanol, and nonanol. Dehulled peas and pea proteins contain a higher number of volatiles originating from free fatty acid breakdown than whole peas, which suggests that process transformation and storage after transformation are favourable to enzymatic oxidation and autoxidation. Indeed, dehulling exposes lipids or free fatty acids to more oxygen and promotes lipid degradation [37]. Hulls contain phenolic compounds with antioxidant properties that limit oxidation phenomena [37,52].

Butanol and pentanol are the two major alcohols identified in chickpeas [43].

Low-tannin faba beans contain higher relative percentages of ethanol, 3-methylbutanol, and 2-methylbutanol than those of high-tannin faba beans. These volatiles come from amino acid degradation, and a supposition is that low-tannin cultivars contain more spoilage microorganisms or have an important secondary metabolism (the Ehrlich−Neubauer pathway). Indeed, low-tannin cultivars are less resistant to pathogens that can promote the implantation of unwanted microorganisms [53]. Concerning the faba bean location, no difference is observed between the two conditions. During storage of faba beans, the relative percentages of alcohols decrease for each stored temperature.

### 4.5. Ketones

Ketones (Table 7) constitute an intermediate class of volatiles except for dehulled peas and pea protein isolates (wet process), which contain a high percentage of ketones. Ketones come from amino acid degradation, free fatty acid oxidation, and carotenoid breakdown, while free fatty acid oxidation is predominant. CDC Rio and Onyx contain a higher percentage among the black beans; moreover, AC Pintoba and Maverick have a high percentage of ketones [40]. In whole peas, the 2006 cultivar presents a higher percentage than those of the 2005 and 2007 seeds. Thus, dehulling exposes lipids to more oxygen and limits the presence of antioxidants that promote the production of volatiles [37]. Dehulled peas contain more ketones than whole peas, and this phenomenon also occurs in whole faba beans and after dehulling [11,37]. Pea protein isolates have eight times more ketones than dehulled peas, with a high presence of 2,3-pentanedione, 5-hexen-2-one, heptanone, 2-methyl-3-heptanone, 3-octen-2-one, and 2,3-octanedione [42]. These compounds are derived from free fatty acid breakdown and could be explained by an increase in temperature during the wet process that promotes autoxidation. No difference in the relative percentage is highlighted in the two chickpea cultivars, faba bean tannins, and location [37,41,43]. However, during storage for a short time period, ketones decrease in comparison with flour before storage [45]. For example, 6-methyl-5-hepten-2-one, which comes from free fatty acid breakdown, disappears at refrigerated and frozen temperatures but increases when stored at room temperature. This observation shows that low temperatures limit the enzymatic activity of LOX or autoxidation. Control of storage is an important parameter to avoid off-notes in pulses.

### 4.6. Acids

Organic acids (Table 8) are only identified in dehulled peas, pea proteins, chickpeas, faba beans (tannins), and faba beans (storage). Acetic acid, 2-methylbutanoic acid, and 3-methylbutanoic acid come from free amino acid degradation (the Ehrlich−Neubauer pathway), whereas longer-chain acids, such as hexanoic, octanoic, and nonanoic acids, originate from free fatty acid degradation. Palmitic and oleic acids are naturally present in pulses, but these lipids are stored as triglycerides or phospholipids, and their release is due to the action of lipases [17,19].

Dehulled peas and pea protein concentrates have the same percentage of 3-methylbutanoic acid (rancid and cheese-like odour [50]). In contrast, dehulled peas and pea protein isolates present similar percentages of 2-ethyl hexanoic acid and nonanoic acid (fatty odour [50]). Chickpeas contain the largest relative percentage of acetic acid (vinegar-like odour [50]) compared to faba beans (tannins) and faba beans (storage), whereas 3-methylbutanoic acid is predominant in faba beans (tannins and storage). These differences could be explained by variations in the amino acid composition or by different metabolic activities in the plant or in spoilage microorganisms [26]. Concerning faba beans (storage), for each acid, the peak area percentage is equal to unstored flour and stored flours at refrigerated and frozen temperatures, but flour stored at room temperature shows a low peak area percentage [45]. Data given as relative percentages suggests that the difference could be explained by a high increase in the aldehyde peak area for room temperature flour that decreases the relative percentage of the acid peak area.

### 4.7. Esters

Esters (Table 9) represent a minor class of volatiles in pulses except for pea protein isolates and stored faba beans. The origins of only three volatiles have been identified: methyl-3-methyl butanoate is derived from amino acids, whereas ethyl ethanoate and hexyl hexanoate come from free fatty acids. Pea protein isolates (wet process) have a 53 times greater concentration of esters than dehulled peas with the predominance of ethyl propanoate [42]. In contrast, octyl hexanoate is the most important ester identified in pea protein concentrates (dry process) [44]. For chickpeas, the Kabuli cultivar has a higher relative percentage than that of Desi and contains more 5-isobutylnonane and 4-dodecanoyloxybutyl dodecanoate [43]. During short storage of faba beans, the relative percentage of esters increases [45]. Butyl ethanoate, hexyl ethanoate, octyl 2,2-dimethylpropanoate, hexyl 2-methylbutanoate, ethylbutanoate, 2-ethylhexyl butanoate, isopentyl isopentanoate, butyl hexanoate, hexyl hexanoate, and methyl salicylate appear only at refrigerated and frozen temperatures.

### 4.8. Pyrazines

Pyrazines (Table 10) are only identified in the pea samples (except for the pea protein concentrate). Pyrazines are produced from amino acids and carbohydrates at room temperature (acyloin degradation) and high temperatures (the Strecker degradation) [42].

### 4.9. Terpenes

Terpenes present in pulses are composed of monoterpenes (C_10_) and sesquiterpenes (C_15_) (Table 11), and most of them come from the degradation of carotenoids. Limonene (citrus aroma [50]) is the most common terpene identified in pulses and appears in faba beans during storage. Its percentage is more important in flour stored at frozen temperature than at refrigerated temperature [45]. In common beans (black beans, pinto beans, and dark red kidney beans), α-pinene (fresh herbal and tropical fruit notes [50]), Δ3-carene (pungent, turpentine-like taste [50]), and limonene are detected. Among the common beans, AC Harblack (black beans) and dark red kidney bean cultivars have the lowest content of terpenes, while CDC Rio and Onyx cultivars for black beans have approximatively the same content as the Maverick cultivar (pinto beans). AC Pintoba (pinto beans) has the greatest content of terpenes (16 times more than AC Harblack). Dehulled peas contain more terpenes in number and content than whole peas and pea proteins. t-Muurolol, torreyol, and elemol are the predominant terpenes in dehulled peas. Menthol is only present in faba beans (tannins and storage).

### 4.10. Furans

Currently, free fatty acid degradation is the only origin identified for the synthesis of furans (Table 12). No furan was identified in chickpeas [43]. 2-Acetylfuran (coffee-like aroma [50]) is the only furan present in dehulled peas and pea protein isolates [42]. 2-Propylfuran and 2-propionyl furan are only present in common beans (black beans, pinto beans, and dark red kidney beans), whereas the presence of 2-ethylfuran (smoky burnt odour [50]) depends on the cultivar, as this volatile is not detected in AC Harblack (black beans) and ROG 802 (dark red kidney beans) [41]. For whole peas, the 2006 Eclipse cultivar contains a higher percentage of 2-methylfuran (chocolate odour [50]) than that of the 2007 cultivar. 2-Methylfuran and 2-ethylfuran are not present in 2005, while the same percentage of 2-ethylfuran is identified in 2006 and 2007 cultivars [11]. As is also observed for hexanal (Table 4), the 2006 cultivar contains more volatiles from free fatty acid degradation with a higher percentage than those of the cultivars from the two other years. It could be supposed that environmental stressors were more important, or the storage conditions were less controlled or longer than for 2005 and 2007.

Protein concentrates contain 2-pentylfuran (green bean, metallic, vegetable odour [50]) [44], and because of the leak of data about pulse volatiles before transformation (high temperature or activity of LOX), no conclusions can be drawn about its impact.

Concerning faba beans, 2-ethylfuran is only identified in faba beans (location), and 2-pentylfuran is present in the 3 conditions of faba beans (tannins, location, and storage). For faba beans (storage), raw flour and frozen-stored flour present a higher percentage of 2-pentylfuran than flours stored at room temperature and refrigerated temperatures [45]. This result is not congruent with previous data that were in line with the limitation of LOX activity in frozen conditions.

### 4.11. Lactones

Lactones (Table 13) are not identified in common beans (black beans, pinto beans, and dark red kidney beans), whole peas, pea protein concentrates, or faba beans (location).

Dehulled peas have a higher relative percentage of lactones than pea protein isolates, while both concentrations are approximately the same (9.87 and 9.72 µg/g of dry matter, respectively) [42]. 3-Methylbutyrolactone and γ-pentalactone (sweet and herbaceous odour [50]) are only present in dehulled peas, whereas 4-hydroxy-2-hexenoic acid lactone is only identified in pea isolates. Dehulled peas contain a high percentage of γ-caprolactone compared to that of pea isolates, and pea isolates are more concentrated in γ-methyl-γ-caprolactone and 4-hydroxy-2-noneic acid lactone. Lactones are naturally present in plants and seem to be derived from oleic acid [28]. During pea protein production, enzymatic oxidation and/or autoxidation of free fatty acids could promote the production of volatile compounds, such as lactones.

Low- and high-tannin faba beans present the same relative percentages of lactones except for γ-butyrolactone (faint and slightly buttery odour [50]), which is two times more concentrated in high-tannin flour [37].

During short storage of faba beans, the relative percentage of γ-butyrolactone decreases more quickly at room temperature than at refrigerated and frozen temperatures [45]. Moreover, the relative percentages of δ-caprolactone and γ-caprolactone are more important for faba beans stored at frozen temperatures. Low temperatures limit the formation of volatiles because they block enzymatic activities and do not provide the necessary energy for degradation reactions. Because the data are expressed as relative percentages, they do not reflect the real concentrations of volatiles and could result in false interpretations.

### 4.12. Other Volatiles

Other volatiles (Table 14) are mainly present in common beans (black beans, pinto beans, and dark red kidney beans), whole peas, and chickpeas. They do not belong to a specific class of volatiles, and their origin has not been studied.

Pulses present different compositions of free fatty acids and amino acids that induce a high variety of volatile compounds. Different factors, such as environmental stress conditions in fields, uncontrolled storage, transformation of seeds (dehulling, production of proteins), and lipoxygenase activity, promote the production of volatiles. Moreover, cultivars have a different nutritional composition, which could explain the difference in the percentage of volatiles. For example, high-tannin faba beans contain more lipids that promote the production of volatiles from free fatty acids [37]. Most of these volatiles have an unpleasant odour or aroma that contributes to pulses’ off-flavours. Moreover, some articles did not mention all the details about pulses, such as crop year, environmental field conditions, cultivar, or storage conditions, before analysis. These details are important and could explain the origin of the differences. Finally, it is important to highlight the odour-active compounds among all the volatiles present in pulses to determine adequate strategies to reduce their perceptions. Odour-active volatiles are identified by GC-Olfactometry (GC-O).

## 5. Odour-Active Compounds in Pulses

An odour-active compound is a volatile compound whose concentration is greater than or equal to its odour detection threshold. It contributes to the product’s aroma. These compounds can be detected by GC-O.

Although many volatiles have been identified in pulses, their odour-active potential is rarely highlighted. GC-O analyses were conducted on dehulled seeds and protein isolates of pea [42], and whole [54] and dehulled lupin seeds [28]. The methods used to select volatile compounds involved in the product’s aroma are different according to the studies. For whole peas and pea protein isolates, the results are given as the Percentage of Detection (%D), which represents the number of panellists that detected volatile compounds over the total number of panellists [42]. For whole lupins, the duration and intensity (from 0 to 10) of each detected volatile enabled the authors to calculate the area under the curve. The peak area of each compound corresponds to the Odour Intensity (OI) [54]. Concerning dehulled lupins, Aroma Extract Dilution Analysis (AEDA) was used, and the data are given as the Factor Dilution (FD) [28]. The extract is diluted several times (from 0 to 2048), and panellists have to determine if they detect volatiles; a high FD is correlated to a high concentration of the volatile in the sample and/or to a weak threshold detection. The list of odour-active compounds identified in these pulses is presented in Table 15. 

In dehulled peas, 15 odour-active compounds are present compared to 16 in pea protein isolates, and they are related to different types of odour descriptors, such as mushroom/earth, vegetable/green/pea, empyreumatic (peanut-grilled), animal, floral/fruity, and sweet [42]. For lupin, 19 odour-active compounds (including an unknown volatile) were identified in whole grains and 26 in dehulled grains. These volatiles are described as sweet, beany/pea/green/cardboard, floral, mushroom, fruity/floral, and animal [28,54]. Odour-active compounds are distributed into aldehydes, alcohols, ketones, acids, lactones, and pyrazines; however, aromatic hydrocarbons, alkanes, alkenes, esters, terpenes, and furans are not involved in the aroma of these pulses. The origin of these compounds is free fatty acid oxidation, except for acids and pyrazines, which are derived primarily from amino acid degradation.

Dehulled peas and pea isolates share the following odourant compounds: vanillin, hexanal, (E,E)-2,4-decadienal, 1-octen-3-ol, 2,3-octanedione, undecanone, γ-octalactone, 4-hydroxy-2-noneic acid lactone, 5- or 6-methyl, and benzothiazole. Some compounds are not detected in whole peas but are active compounds in pea protein isolates, such as 4-ethybenzaldehyde, phenylacetaldehyde, 3-methylthiopropanal, heptanal, (E)-2-octenal, (E,E)-3,5-octadien-2-one, heptanoic acid, and dimethyl trisulfide; they mainly come from free fatty acids and amino acids. However, nonanal, pentanol, octanol, nonanol, 3-methylbutanoic acid, and γ-nonalactone are only active in dehulled peas; they mainly come from free fatty acid oxidation. Curiously, nonanal, pentanol, and octanal are more concentrated in volatile extracts of pea protein isolates than dehulled peas but are only perceived as odour-active in dehulled samples. The authors suggested that the threshold hypothesis could not be applied without more explanations [42].

Whole and dehulled lupins have only a few odour-active compounds in common: (E)-2-nonenal, (E,Z)-2,6-nonadienal, 1-octen-3-one, 3-isobutyl-2-methoxypyrazine, and 3-isopropyl-2-methoxypyrazine. Many compounds from amino acids are only present in dehulled lupin, such as 2-acetyl-1-pyrroline, maltol, sotolone, and some acids. They are probably due to contamination by microorganisms in these grains. Nevertheless, numerous odour-active compounds present only in whole lupins come from free fatty acid degradation. This difference between the two samples could be due to the storage conditions, the cultivar choice, or the transformation step (dehulling).

Vanillin and γ-octalactone are odour-active compounds in peas and dehulled lupins. γ-Octalactone is described as floral/anise/mint in peas and coconut/sweet in lupins. Hexanal is not an odourant only in dehulled peas. Pea protein isolates and whole lupins have two odourant compounds in common: (E)-2-octenal and dimethyl trisulfide; this last compound is described as faeces/meat broth/sewer in peas and meaty/metallic/sulphur in lupins. 3-Methylbutanoic acid and γ-nonalactone are odour-active compounds of whole peas and dehulled lupins; 3-methylbutanoic acid is perceived as animal in peas but sweaty/fruity/cheesy in lupins. These differences are not necessarily due to the variety of pulses; the descriptors depend on the panellists, the possible presence of coelution, or large differences in the volatile concentrations.

Odour-active compounds present in peas and lupins are derived mainly from free fatty acids and amino acids. The large variety of these perceived volatiles depends on the type of pulses, the cultivar, the storage conditions, and the transformation steps. Finally, descriptors for the same molecule could be very different and depend on the studied matrix.

## 6. Conclusions and Perspectives

This review constitutes a compilation of the different volatile compounds identified in pulses with a focus on those that are odour-active and a discussion on their potential origins to reduce the off-notes.

A qualitative comparison of volatile compounds between pulses is presented. Indeed, the main drawback to making quantitative comparisons of volatiles is that the data are often expressed as peak areas or relative percentages, without using a standard compound allowing real quantification of each volatile compound. However, it was possible to suggest some hypotheses on the origins of the off-notes present in pulses with the aim of increasing the acceptability of pulses in food for humans.

The diversity of unsaturated free fatty acid contents and the characteristics of endogenous lipoxygenases in pulses mainly explain the large variety of volatile compounds identified. All of the classes of volatiles are present in pulses, but ketones, pyrazines, furans, and lactones are minority classes. Aromatic hydrocarbons represent more than 38% of the volatiles detected in common beans (black beans, pinto beans, and dark red kidney beans), whereas aldehydes and alcohols are more specific to dehulled peas, pea proteins (concentrate and isolate), and faba beans (location and storage). Seed transformations or uncontrolled parameters of storage promote the generation of volatile compounds from the degradation of free fatty acids and amino acids. For whole peas, the 2006 harvest-year seeds present higher percentages of aromatic hydrocarbons, aldehydes, alkanes, ketones, and furans than those of the 2005 and 2007 seeds [11]. If the storage conditions are supposed to be equivalent for these three harvest years, bad culture conditions could be suggested during 2006, such as water stress and/or mechanical or insect attacks, compared to years 2005 and 2007 and could explain these differences in terms of volatiles. Finally, all of these parameters could account for such heterogeneity in volatile compounds, yet they are not always taken into consideration in explaining the variability in aromatic composition.

Although the number of volatile compounds identified in pulses is high, only a small part contributes to the aroma. These odour-active compounds present different descriptors and threshold detection. However, identifying odour-active compounds in pulses is rarely done, and only two studies report on their olfactory impact, namely in lupins and peas. Only aldehydes, alcohols, ketones, acids, lactones, and pyrazines are involved in the aroma. The origin of these compounds is free fatty acid oxidation, except for acids and pyrazines, which originate primarily from amino acid degradation by unwanted microorganisms. Off-notes are described as vegetable, green, hay, potato, bean, metallic, mushroom, animal, dust, solvent, cardboard, etc., and refer to “beany” notes, but other volatiles present a pleasant smell, such as floral, fruity, grilled, sweet, and vanilla odours.

Precise identification of the odour-active compounds in pulses could allow the determination of their main origins and the proposal of strategies to reduce their perception. Some strategic axes have been identified to improve pulses’ aroma: to limit the production of volatile compounds, to remove the off-notes, and to decrease the perception of off-notes [6,56]. One approach consists of generating lipoxygenase-free legume seeds to limit off-note production, and this experiment was carried out on soybeans [57]. However, volatiles provided by the LOX pathway are also fully involved in a mechanism of defence for plants [8]. Therefore, a potential lower resistance of mutated plants should be considered. Moreover, due to climate change, water stress, or other stressors increase in future years, these new cultivars could be less adapted and produce lower yields. Moreover, heat treatments, such as blanching, microwave, radiofrequency, and conventional heating of mature seeds, allow the decrease or inhibition of lipoxygenase activity [58,59]. However, high temperatures favour the autoxidation of unsaturated free fatty acids. A compromise must be considered to limit these two phenomena. Finally, for the past 20 years, fermentation has modified the aroma profile of pulses by reducing or masking off-notes. Some molecules are still detected, such as pentanal, hexanal and heptanal in fermented lupin proteins or 2,3-butanedione, hexanal, 1-penten-3-one,2-heptanone, and 3-methylbutanol in fermented pea proteins [60,61]. A perspective that must be studied is the use of microorganism coculture to improve the aroma of pulses [62]. Another approach could consist of using perceptual interactions to mask off-notes or modify the aroma product to a pleasant product, in particular using odour-mixture, odour-taste, and/or odour-texture interactions [63,64].

Furthermore, non-volatile compounds also contribute to the off-flavours of pulses, particularly for bitterness and astringency (off-tastes). Saponins, alkaloids, and phenolic compounds have been identified in pulses, but their role in sensorial perception has rarely been studied. The elimination of these molecules could increase the acceptability of plant-based products.

## Figures and Tables

**Table 1:** Production of aldehydes, alcohols, and acids from some amino acids in pulses (adapted with permission from [26]; Copyright (2021) CRC Press).

Amino Acids	Aldehydes	Alcohols	Acids
Alanine	Acetaldehyde	Ethanol *	Acetic acid *
Phenylalanine	Phenylacetaldehyde *	2-Phenylethanol *	Phenylacetic acid
Phenylalanine	Benzaldehyde *	Benzyl alcohol *	Benzoic acid
Valine	2-Methylpropanal *	2-Methylpropanol *	2-Methylpropanoic acid
Methionine	3-Methylthiopropanal *	3-Methylthiopropanol	3-Methylthiopropanoic acid
Isoleucine	2-Methylbutanal *	2-Methylbutanol *	2-Methylbutanoic acid *
Leucine	3-Methylbutanal *	3-Methylbutanol *	3-Methylbutanoic acid *

* Volatile compounds identified in the studied pulses.

**Table 2:** Characteristics of pulses: extraction and identification methods of volatile compounds.

Code	Pulses	Cultivar	Year	Location	Storage		Seed Transformation	Extraction	Separation and Identification	References
**Black bean**	Black bean (*Phaseolus vulgaris* L.)	AC Harblack	2005	Morden, Canada	Dry room (23 °C, 15–20% RH) (whole).		Ground in flour (coffee mill) (whole).	HS-SPME: 10 g in a 125-mL Erlenmeyer flask capped, DVB/CAR/PDMS Stable Flex SPME fibre at 50 °C for 1 h.	GC-MS: desorption at 250 °C for 2 min, Supelcowax 10 polar column, started at 40/1/70 °C, then 70/5/200 °C and 200/50/250 °C.	[40]
		CDC Rio								
		Onyx								
**Pinto bean**	Pinto bean (*Phaseolus vulgaris* L.)	AC Pintoba								
		Maverick								
**Dark red kidney bean**	Dark red kidney bean (*Phaseolus vulgaris* L.)	ROG 802								
		Redhawk								
**Whole pea**	Pea (*Pisum* *sativum* L.)	Eclipse (Yellow field pea)	2005	Near Saskatoon, Canada	4 °C (whole).		Ground in flour (whole).	HS-SPME: 3 g, CAR/PDMS SPME Fibre at 50 °C for 30 min.	GC-MS: desorption at 300 °C for 3 min, VF-5MS capillary column, started at 35/6/80 °C and 80/20/280 °C.	[11]
			2006							
			2007							
**Dehulled pea**		-	Before 2013	-	In a glass bottle, −18 °C.		Ground in flour (dehulled).	SAFE: 20 g in 100 mL of water, 2 h at 30 °C and 10–2 mbar. Liquid-liquid separation with 3 × 10 mL of CH2Cl2. Concentration using Kuderna Danish apparatus, 70 °C.	GC-MS: ZB1.MS non-polar column, injection of 2 µL, started at 50/4/160 °C, then 160/15/320 °C.	[42]
**Pea protein**							Protein isolate, Nutralys^®^ (dehulled, wet process).			
		-	Before 2020	-	-		Protein concentrate (dehulled, dry process).	HS-SPME: 1.5 g was dissolved into saturated NaCl solution for 1 h at 20 °C, then transferred into a bottle and incubated at 50 °C in an ultrasonic bath, insertion of the DVB/CAR/PDMS Stable Flex SPME fibre at 50 °C for 20 min.	GC-MS: desorption at 250 °C for 3 min, DB-5MS column, started at 40/5/70 °C, then 70/10/200 °C and 200/50/250 °C.	[44]
**Chickpea**	Chickpea (*Cicer* *arietinum*)	Kabuli (Benying-1)	2018	Urumqi, China	−18 °C for a maximum of 3 weeks (whole). −20 °C for a maximum of 1 week (powder).		Dried using sunlight before storage (whole). Ground to a fine powder (80 mesh, mill).	HS-SPME: 1.5 g was dispersed in water and 5 mL was placed in a 20-mL headspace sampling vial and capped, PDMS/DVB fibre at 60 °C for 60 min.	GC-MS: desorption at 250 °C for 5 min, PEG 20 M column, started at 35/5/130 °C and 130/9/200 °C.	[43]
		Desi (YZ-364)								
**Faba bean Tannin**	Faba bean (*Vicia faba* L. *minor*)	High tannin	2016	Alberta, Canada	In freezer bags (polypropylene), 22 °C and 18% RH in a dark and solvent-free room (flour).		Ground in flour (impact mill) (whole).	HS-SPME: 2 g was pre-incubated at 50 °C for 5 min, DVB/CAR/PDMS Stable Flex SPME fibre at 50 °C for 1 h.	GC-MS: desorption at 250 °C for 60 s, DB-17 mod polarity column, 40/5/200 °C.	[37]
		Low tannin								
**Faba bean Location**		Low tannin (13 cultivars)	2009	Barrhead, Canada	-		Ground in flour (coffee mill) (whole).	HS-SPME: 10 g in a 125-mL Erlenmeyer flask capped, DVB/CAR/PDMS Stable Flex SPME fibre at 50 °C for 1 h.	GC-MS: desorption at 250 °C for 2 min, Supelcowax 10 polar column, started at 40/1/70 °C, then 70/5/200 °C and 200/50/250 °C.	[41]
				Namao, Canada						
**Faba bean Storage**		High tannin	2016	Alberta, Canada	No storage.		Ground in flour (micro-mill) with a water-cooled system to protect from overheating (whole).	HS-SPME: 2 g was pre-incubated at 50 °C for 5 min, DVB/CAR/PDMS Stable Flex SPME fibre at 50 °C for 1 h.	GC-MS: desorption at 250 °C for 60 s, DB-17 mod polarity column, 40/5/200 °C.	[45]
					In bags (PE), in a dark and solvent-free room, 60 days (flour).	22 °C, 19% RH.				
						4 °C, 9% RH.				
						−21 °C.				

RH, relative humidity; SAFE, Solvent-Assisted Flavour Evaporation; HS-SPME, HeadSpace Solid-Phase MicroExtraction; PE, polyethylene.

**Table 3:** Aromatic hydrocarbons in pulses (expressed as percentages).

Aromatic Hydrocarbons	CAS	Origin (s)	Black Bean	Pinto Bean	Dark Red Kidney Bean	Pea		Chickpea	Faba Bean		
						Whole	Dehulled		Tannin	Location	Storage
Toluene	108-88-3	FFA ^1,2^	0.00–0.86	0.96–1.86	0.00–0.73	1.20–2.40	Coelution		0.26–0.37	0.88–0.96	0.41–3.12
m-Ethyltoluene	620-14-4								0.09–0.11		0.00–0.28
Benzene	71-43-2	FFA ^4^				0.00–0.50			0.09–0.11		
Ethylbenzene	100-41-4	FFA ^1,2^	0.00–0.45	0.00–0.44		0.30–0.80			0.12–0.17	1.19–1.28	0.19–0.73
1,2,3-Trimethylbenzene	526-73-8	FFA ^2^	0.00–0.54	0.54–1.03					0.12–0.13		0.00–0.21
1,3,5-Trimethylbenzene	108-67-8	FFA ^2^	0.00–0.77	0.81–1.84							0.00–0.19
Propylbenzene	103-65-1		0.00–0.35	0.00–0.40							0.00–0.19
Cumene	98-82-8	FFA ^2^	0.70–1.13	0.84–0.86	1.11–1.69	0.30–0.60				0.42–0.46	
p-Xylene	106-42-3	FFA ^1^	1.15–1.50	0.00–1.20	0.00–1.17	0.40–1.00				0.27–0.38	0.00–0.70
o-Xylene	95-47-6	FFA ^1,2,4^	0.95–1.16	1.14–1.73	1.13–1.45		Coelution				
m-Xylene	108-38-3	FFA ^1^					1.25				
4-Ethyl-m-xylene	874-41-9								0.05–0.07		0.00–0.64
p-Cymene	99-87-6						Trace		0.32–0.33		
Styrene	100-42-5	FFA ^1,2,3^; N ^2,3^	32.55–45.47	30.88–31.76	38.75–47.57	0.70–2.20		0.00–0.60		11.36–13.79	0.00–1.90
α-Methylstyrene	98-83-9		0.00–0.43	0.00–0.38	0.00–0.49						
**Total**			38.51–49.28	38.24–38.51	43.41–50.72	3.50–6.70	1.25	0.00–0.60	1.07–1.27	14.15–16.89	0.68–7.75

^1^ [11]; ^2^ [40]; ^3^ [41]; ^4^ [14,21]. FFA, free fatty acids; N, naturally present (not considered as a contaminant). The value “min–max” corresponds to the minimum and maximum percentages for each volatile compound identified. “Black bean” refers to the 3 cultivars studied, and “Pinto bean” and “Dark red kidney bean” correspond to 2 cultivars for each [40]. For peas, “Whole” corresponds to 3 different harvest years of the cultivar Eclipse (2005, 2006, and 2007) [11], and “Dehulled” corresponds to dehulled pea flour [42]. “Chickpea” refers to 2 cultivars [43]. For faba beans, “Tannin” corresponds to a group of low-tannin cultivars and a group of high-tannin cultivars [37], “Location” corresponds to different low-tannin cultivars that were harvested at 2 different locations in Canada [41], and “Storage” corresponds to high-tannin cultivars that were stored under 3 different conditions (ambient, positive, and negative temperatures) for 60 days and a control (sample stored for 0 days) [45]. No aromatic hydrocarbon was detected in pea proteins (concentrate and isolate) (see Table 2 for more details).

**Table 4:** Aldehydes in pulses (expressed as percentages).

Aldehydes	CAS	Origin (s)	Black Bean	Pinto Bean	Dark Red Kidney Bean	Pea			Chickpea	Faba Bean		
						Whole	Dehulled	Proteins		Tannin	Location	Storage
Benzaldehyde	100-52-7	FFA ^2^; AA ^5,7,11^	2.36–3.64	2.58–2.60	3.46–4.04			0.00–2.11	0.00–2.19	0.36–0.45	2.09–2.10	0.46–0.85
4-Ethylbenzaldehyde	53951-50-1							0.00–0.54				
Phenylacetaldehyde	122-78-1	AA ^3,5,7,11^						0.00–0.67		0.14–0.17		0.21–0.39
Vanillin	121-33-5						0.16	0.00–0.08				
2-Methylpropanal	78-84-2	AA ^11^								0.49–0.51		
3-Methylthiopropanal	3268-49-3	AA ^3,11^						0.00–0.03				
2-Methylbutanal	96-17-3	AA ^5,8,11^						0.03–0.49		0.31–0.48		0.00–0.13
3-Methylbutanal	590-86-3	AA ^5,7,11^				0.00–1.00		0.00–0.10		1.04–1.17		0.36–0.59
Pentanal	110-62-3	FFA ^2,9,10^	0.00–0.59	0.60–0.79	1.03–1.11			0.00–0.73			0.96–1.21	
Furfural	98-01-1							0.00–0.05				
Hexanal	66-25-1	FFA ^1,2,3,4,6,7,8,9,10^	12.76–16.71	9.77–11.27	15.88–18.6	1.50–6.10	0.93	27.22–54.12		10.28–13.85	40.78–40.88	1.29–25.07
2-Ethylhexanal	123-05-7											0.00–0.16
(E)-2-Hexenal	6728-26-3	FFA ^1,2,4,8,9,10^	0.00–1.60	0.00–1.67	0.00–1.75			0.00–0.19				
Heptanal	111-71-7	FFA ^2,3,7,8,9,10^	0.00–0.75	0.00–0.82	0.00–0.95			1.13–5.31		1.04–1.14	1.10–1.14	0.00–1.91
(E)-2-Heptenal	18829-55-5	FFA ^1,2,4,8,9,10^	1.52–1.75	1.54–2.01	1.52–1.88	0.00–2.60		0.00–0.58			1.79–1.97	
(E,E)-2,4-Heptadienal	4313-03-5	FFA ^2,8,9,10^	0.57–0.66	0.57–0.59	0.00–0.71		Trace	0.00−Coelution				
Octanal	124-13-0	FFA ^2,7,8,9,10^	0.00–0.62		0.00–0.68			0.00–0.58	1.35–1.76	2.07–2.40	1.15–1.29	0.08–2.22
(E)-2-Octenal	2548-87-0	FFA ^1,3,7,8,9,10^				3.00–13.10		0.00–0.10	2.13–2.16	0.23–0.23	0.48–0.51	0.19–0.45
Nonanal	124-19-6	FFA ^2,4,6,7,8,9,10^	2.11–3.08	2.42–2.80	2.13–3.60		1.30	3.12–5.24	3.70–4.60	9.8–12.31	7.99–10.54	3.54–36.29
(E)-2-Nonenal	18829-56-6	FFA ^2,7,9,10^	0.00–0.67	0.00–0.58						0.20–0.26		0.14–2.52
4-Oxononanal	74327-29-0								0.72–0.91			
(E,E)-2,4-Nonadienal	5910-87-2	FFA ^7,9,10^							0.00–0.44			
Decanal	112-31-2	FFA ^2,7,10^	0.81–1.59	0.00–1.06	0.72–0.99		Coelution	Coelution−1.33	1.28–1.33	2.86–3.26	1.15–1.41	0.37–6.16
(Z)-2-Decenal	2497-25-8	FFA ^7,10^							0.79–1.15			
(E,E)-2,4-Decadienal	25152-84-5	FFA ^3,7,9,10^					Trace					
Undecanal	112-44-7									0.22–0.26		0.00–1.61
Dodecanal	112-54-9											0.00–0.89
Tetradecanal	124-25-4						0.63		0.26–0.55			0.00–0.39
**Total**			24.42–28.01	18.19–23.57	25.77–33.33	10.70–17.70	3.02	40.21–63.66	12.41–12.91	29.14–36.39	58.05–60.58	9.19–79.03

^1^ [11]; ^2^ [40]; ^3^ [42]; ^4^ [44]; ^5^ [37]; ^6^ [41]; ^7^ [45]; ^8^ [35]; ^9^ [25]; ^10^ [14,21]; ^11^ [26]. FFA, free fatty acids; AA, amino acids. The value “min–max” corresponds to the minimum and maximum percentages for each volatile compound identified. “Black bean” refers to the 3 cultivars studied, and “Pinto bean” and “Dark red kidney bean” correspond to 2 cultivars for each [40]. For peas, “Whole” corresponds to 3 different harvest years of the cultivar Eclipse (2005, 2006, and 2007) [11], “Dehulled” corresponds to dehulled pea flour [42], and “Protein” refers to protein concentrate (dry process) [44] and protein isolate (wet process) [42]. “Chickpea” refers to 2 cultivars [43]. For faba beans, “Tannin” corresponds to a group of low-tannin cultivars and a group of high-tannin cultivars [37], “Location” corresponds to different low-tannin cultivars that were harvested at 2 different locations in Canada [41], and “Storage” corresponds to high-tannin cultivars that were stored under 3 different conditions (ambient, positive, and negative temperatures) for 60 days and a control (sample stored for 0 days) [45] (see Table 2 for more details).

**Table 5:** Alkanes/alkenes in pulses (expressed as percentages).

Alkanes/Alkenes	CAS	Origin (s)	Black Bean	Pinto Bean	Dark Red Kidney Bean	Pea			Chickpea	Faba Bean		
						Whole	Dehulled	Proteins		Tannin	Location	Storage
Trichloromethane	67-66-3	N ^1^				0.00–0.50						
Octylcyclopropane	1472-09-9		0.00–0.96	0.00–1.28	0.00–2.04							
Pentane	109-66-0	FFA ^4^								0.10–0.10		
Hexane	110-54-3	FFA ^4^	0.74–1.54	1.14–1.72	0.83–0.86							
3-Methylhexane	589-34-4					0.00–2.50						
Butylcyclohexane	1678-93-9		0.00–0.49	0.00–0.51								
Heptane	142-82-5	FFA ^3^	0.54–1.15	0.94–1.07	0.57–0.76						0.55–0.56	
Octane	111-65-9	FFA ^4^	1.74–2.66	0.00–1.74	1.89–3.64						1.79–1.96	
2,6-Dimethyloctane	2051-30-1		0.00–0.48	0.46–0.83								
Nonane	111-84-2	FFA ^4^	0.75–0.95	1.00–1.37	0.77–1.17					0.16–0.17	0.34–0.36	
3-Methylnonane	5911-04-6		0.00–0.52	0.56–0.80								
4-Methylnonane	17301-94-9		0.00–0.94	0.95–1.64								
3,7-Dimethylnonane	17302-32-8									0.18–0.19		
Decane	124-18-5	FFA ^2,4^	2.66–4.27	4.78–6.55	1.60–1.86					0.67–0.70		0.95–3.88
4-Methyldecane	2847-72-5		0.00–0.75	0.64–1.34								
2,4-Dimethyldecane	2801-84-5											0.00–0.32
3,7-Dimethyl-decane	17312-54-8								0.00–0.31			
Undecane	1120-21-4	FFA ^1,4^	0.00–0.87	1.06–1.51		1.80–2.60				0.15–0.15	1.36–1.52	
Dodecane	112-40-3		0.00–1.12	1.22–1.27		0.80–3.60				0.57–0.62		0.80–3.22
2,4-Dimethyldodecane	6117–99–3	FFA ^1^								0.09–0.09		0.00–0.33
5,8-Diethyldodecane	24251-86-3									0.04–0.06		
2,6,10-Trimethyldodecane	3891-98-3		0.00–0.79	0.64–1.27								
Tridecane	629-50-5					0.90–4.30				0.06–0.08		0.00–0.15
2-Methyltridecane	1560-96-9	FFA ^1^								0.05–0.05		0.00–0.14
3-Methyltridecane	6418-41-3	FFA ^1^								0.12–0.15		0.00–0.42
2,2-Dimethyltridecane	61869-04-3									0.13–0.15		
Tetradecane	629-59-4					0.00–1.10	Trace	0.00–1.10		1.30–1.90		0.48–1.34
Pentadecane	629-62-9							0.00–1.57		0.19–0.21		0.00–0.64
3-Methylpentadecane	2882-96-4									0.15–0.26		0.00–0.08
Hexadecane	544-76-3							0.00–2.75		0.24–0.26		0.00–0.35
Heptadecane	629-78-7						1.27			0.07–0.10		
Nonadecane	629-92-5									0.02–0.06		0.46–0.99
Tetracosane	646-31-1								0.00–0.57			
Total alkanes			7.84–15.66	16.43–20.00	7.95–8.09	7.30–14.30	1.27	0.00–5.44	0.31–0.57	4.37–5.22	4.06–4.41	4.61–9.22
(E)-5-(Pentyloxy)-2-pentene	34061-80-8							0.00–0.69				
(Z)-1-Methoxy-3-hexene	70220-06-3								0.00–1.91			
3-Ethyl-2-methyl-1,3-hexadiene	61142-36-7							0.00–0.05			0.37–0.42	
1-Tetradecene	1120-36-1						5.74	0.00−Coelution		0.07–0.08		
**Total alkenes**							5.74	0.05–0.69	0.00–1.91	0.07–0.08	0.37–0.42	

^1^ [11]; ^2^ [40]; ^3^ [41]; ^4^ [14,21]. N, naturally present (not considered as a contaminant); FFA, free fatty acids. The value “min–max” corresponds to the minimum and maximum percentages for each volatile compound identified. “Black bean” refers to the 3 cultivars studied, and “Pinto bean” and “Dark red kidney bean” correspond to 2 cultivars for each [40]. For peas, “Whole” corresponds to 3 different harvest years of the cultivar Eclipse (2005, 2006, and 2007) [11], “Dehulled” corresponds to dehulled pea flour [42], and “Protein” refers to protein concentrate (dry process) [44] and protein isolate (wet process) [42]. “Chickpea” refers to 2 cultivars [43]. For faba beans, “Tannin” corresponds to a group of low-tannin cultivars and a group of high-tannin cultivars [37], “Location” corresponds to different low-tannin cultivars that were harvested at 2 different locations in Canada [41], and “Storage” corresponds to high-tannin cultivars that were stored under 3 different conditions (ambient, positive, and negative temperatures) for 60 days and a control (sample stored for 0 days) [45] (see Table 2 for more details).

**Table 6:** Alcohols in pulses (expressed as percentages).

Alcohols	CAS	Origin (s)	Black Bean	Pinto Bean	Dark Red Kidney Bean	Pea			Chickpea	Faba Bean		
						Whole	Dehulled	Proteins		Tannin	Location	Storage
Ethanol	64-17-5	AA ^5^ FFA ^9,10,11^								0.48–1.29		0.00–0.42
2-Phenylethanol	60-12-8	AA ^5,7,11^								0.27–0.34		0.00–0.83
2-Butoxyethanol	111-76-2						0.95	0.00–0.88				
2-Phenoxyethanol	122-99-6						Coelution	0.00−Coelution				0.00–0.30
Propanol	71-23-8	AA ^1^ FFA ^9,10^				0.60–1.30				0.48–0.53		0.00–0.64
2-Propanol	67-63-0									0.48–0.57		0.00–0.15
1,2-Propanediol	57-55-6							0.00–0.04				
2-Methylpropanol	78-83-1	AA ^9,11^				0.00–0.50						
1-Methoxy-2-Propanol	107-98-2								0.00–0.28			
2-Phenyl-2-propanol	617-94-7										0.38–0.41	
1-[1-Methyl-2-(2-propenyloxy)ethoxy]-2-propanol	55956-25-7							0.00–0.09				
Butanol	71-36-3	FFA ^9,10^							4.32–4.72			
2-Butanol	78-92-2									1.29–1.38		0.00–1.90
2-Methylbutanol	137-32-6	AA ^5,8,9,11^						0.06–0.34		3.52–4.80		0.38–5.48
3-Methylbutanol	123-51-3	AA ^5,7,8,9,11^	0.00–0.80	0.58–0.86			0.42	0.06–0.22		1.97–2.43		0.12–3.23
3-Phenyl-2-butanol	52089-32-4								0.00–1.23			
2,3-Butanediol	513-85-9									0.39–0.40		0.00–1.72
Pentanol	71-41-0	FFA ^2,3,4,7,8,9,10^	0.00–1.61	0.00–0.62			1.52	1.17–3.89	4.90–4.98	1.28–1.36	1.85–2.29	0.74–1.57
2-Pentanol	6032-29-7	FFA ^9^					0.23			0.18–0.27		0.00–0.46
3-Methyl-3-pentanol	77-74-7						5.34	0.00–0.60				
1-Penten-3-ol	616-25-1	FFA ^2,3,4,8,9,10^	0.99–1.86	1.34–1.88	2.30–2.64		6.87	0.44–5.58				
2-Penten-1-ol	20273-24-9							0.00–3.84				
5-[3-(4-Methoxyphenyl)-2-oxaziridinyl]-1-pentanol	-								0.33–0.90			
Phenol	108–95–2		0.00–0.47	0.31–0.34				0.00−Coelution	0.54–0.70			
Benzyl alcohol	100-51-6	AA ^5,11^	0.00–0.24	0.26–1.11				0.00−Coelution		0.54–0.54		0.58–1.46
Hexanol	111-27-3	FFA ^2,3,4,6,7,8,9,10^	1.58–1.86	1.39–1.60	1.24–1.25		4.54	0.88–9.53		10.64–11.32	3.87–4.29	0.33–31.41
2-Ethylhexanol	104-76-7		0.29–0.45	0.29–0.66	0.00–0.4			0.00–1.39				0.00–9.38
4-Ethylcyclohexanol	4534-74-1									0.15–0.17		
2,3-Dimethylcyclohexanol	1502-24-5											0.00–0.20
1-Hexen-3-ol	4798-44-1						0.29	0.00–0.14				
(Z)-3-Hexen-1-ol	928-96-1	FFA ^8,9^					4.23	0.00–0.29				
(Z)-4-Hexen-1-ol	928-91-6							0.00–0.33				
Heptanol	111-70-6	FFA ^8,9,10^				0.00–1.20	Coelution	Coelution−0.66		0.32–0.36	0.50–0.54	0.00–0.26
2-Heptanol	543-49-7	FFA ^9^				0.00–0.40						
2-Methyl-3-heptanol	18720-62-2						0.72					
3-Methyl-2-heptanol	31367-46-1					0.00–2.40						
2-Hepten-4-ol	4798-59-8								0.00–0.78			
Octanol	111-87-5	FFA ^3,7,8,9,10^	0.00–0.33	0.29–0.61	0.00–0.29	3.7–10.7	1.16	0.82–1.29		0.24–0.28	1.18–1.48	0.35–0.83
3-Octanol	589-98-0	FFA^1^				0.00–1.3						0.00–0.42
1-Octen-3-ol	3391-86-4	FFA ^2,3,4,6,7,8,9,10^	1.18–1.43	1.23–1.60	0.00–3.51		2.61	1.23–1.38		0.35–0.41	3.93–4.00	0.29–1.64
(E)-2-Octen-1-ol	18409-17-1	FFA ^10^						0.00–0.96			0.37–0.41	
(E,E)-3,5-Octadien-2-ol	69668-82-2									0.10–0.14		
Nonanol	143-08-8	FFA ^7^	0.42–1.30	0.92–1.10	0.00–0.43		1.27	0.00–2.69		0.92–1.14	0.28–0.41	0.85–1.74
Decanol	112-30-1									0.22–0.29		
Undecanol	112-42-5						6.18					0.00–0.09
2-Pentadecyn-1-ol	2834-00-6								0.00–0.55			
**Total**			6.13–7.72	8.05–9.04	4.67–7.41	7.40–12.70	36.33	8.22–30.70	11.22–13.01	24.21–27.63	13.00–13.27	4.34–53.01

^1^ [11]; ^2^ [40]; ^3^ [42]; ^4^ [44]; ^5^ [37]; ^6^ [41]; ^7^ [45]; ^8^ [35]; ^9^ [25]; ^10^ [14,21]; ^11^ [26]. AA, amino acids; FFA, free fatty acids. The value “min–max” corresponds to the minimum and maximum percentages for each volatile compound identified. “Black bean” refers to the 3 cultivars studied, and “Pinto bean” and “Dark red kidney bean” correspond to 2 cultivars for each [40]. For peas, “Whole” corresponds to 3 different harvest years of the cultivar Eclipse (2005, 2006, and 2007) [11], “Dehulled” corresponds to dehulled pea flour [42], and “Protein” refers to protein concentrate (dry process) [44] and protein isolate (wet process) [42]. “Chickpea” refers to 2 cultivars [43]. For faba beans, “Tannin” corresponds to a group of low-tannin cultivars and a group of high-tannin cultivars [37], “Location” corresponds to different low-tannin cultivars that were harvested at 2 different locations in Canada [41], and “Storage” corresponds to high-tannin cultivars that were stored under 3 different conditions (ambient, positive, and negative temperatures) for 60 days and a control (sample stored for 0 days) [45] (see Table 2 for more details).

**Table 7:** Ketones in pulses (expressed as percentages).

Ketones	CAS	Origin (s)	Black Bean	Pinto Bean	Dark Red Kidney Bean	Pea			Chickpea	Faba Bean		
						Whole	Dehulled	Proteins		Tannin	Location	Storage
Acetophenone	98-86-2	AA ^5^; FFA ^6^	0.63–0.98	0.53–0.77	0.00–0.82					0.33–0.36		0.00–0.50
p-Isopropylacetophenone	645-13–6							0.00–0.73				0.15–0.57
p-Acetylacetophenone	1009-61-6											0.19–0.29
Acetone	67-64-1	FFA ^5^	0.66–1.47	0.85–1.12	1.16–1.75					1.99–2.46	1.00–1.02	0.41–0.92
Butanone	78-93-3	FFA ^1,2,6^	0.00–0.41	0.00–0.38		0.00–0.50		0.00–0.97		3.20–3.43	0.58–0.67	0.79–4.82
3-Hydroxy-3-methyl-2-butanone	115-22-0							0.00−Coelution				
Pentanone	107-87-9	FFA ^1,6^				0.00–1.20						
3-Pentanone	96-22-0						0.87					
2,3-Pentanedione	600-14-6	FFA ^6^					0.42	0.00–1.05				
Hexanone	591-78-6	FFA ^6^								0.03–0.04		
Cyclohexanone	108-94-1							0.00–0.15				
5-Hexen-2-one	109-49-9						3.29	0.00–1.75				
6-Methyl-5-hepten-2-one	110-93-0	FFA ^2^; CAR ^4^	1.97–2.45	0.94–1.76	1.27–2.44				0.82–0.87	1.08–1.50	1.06–1.09	0.00–1.27
Heptanone	110-43-0	FFA ^3,6^					0.17	0.25–1.03		0.27–0.32	0.46–0.48	0.24–0.35
2-Methyl-3-heptanone	13019-20-0						6.85	0.00–6.50				
Isobutyl-2-heptenone	-							0.00–0.17				
Octanone	111-13-7	FFA ^6^					0.41					
3-Octanone	106-68-3	FFA ^4,6^					Trace					
3-Octen-2-one	1669-44-9	FFA ^5^						0.28–0.83			0.69–0.86	
2,3-Octanedione	585-25-1						Trace	0.00–1.34	1.71–2.00		0.52–0.53	
(E,E)-3,5-Octadien-2-one	30086-02-3	FFA ^2,5^	1.14–1.98	1.31–2.41	1.42–1.46			0.00–8.00		0.20–0.28		0.16–0.20
Nonanone	821-55-6									0.26		
Decanone	693-54-9							0.00–0.35				
1,6-Dioxacyclododecane-7,12-dione	777-95-7								0.00–0.72			
Undecanone	112-12-9						Trace	0.00–0.18				
2-Butyl-1,3,2-dioxaborinan-4-one	33823-94-8								0.00–0.37			
**Total**			4.95–6.90	4.74–5.38	4.73–5.62	0.50–1.20	12.01	1.69–21.96	3.24–3.25	7.59–8.42	4.48–4.53	3.72–7.46

^1^ [11]; ^2^ [41]; ^3^ [45]; ^4^ [35]; ^5^ [25]; ^6^ [14,21]. AA, amino acids; FFA, free fatty acids; CAR, carotenoids. The value “min–max” corresponds to the minimum and maximum percentages for each volatile compound identified. “Black bean” refers to the 3 cultivars studied, and “Pinto bean” and “Dark red kidney bean” correspond to 2 cultivars for each [40]. For peas, “Whole” corresponds to 3 different harvest years of the cultivar Eclipse (2005, 2006, and 2007) [11], “Dehulled” corresponds to dehulled pea flour [42], and “Protein” refers to protein concentrate (dry process) [44] and protein isolate (wet process) [42]. “Chickpea” refers to 2 cultivars [43]. For faba beans, “Tannin” corresponds to a group of low-tannin cultivars and a group of high-tannin cultivars [37], “Location” corresponds to different low-tannin cultivars that were harvested at 2 different locations in Canada [41], and “Storage” corresponds to high-tannin cultivars that were stored under 3 different conditions (ambient, positive, and negative temperatures) for 60 days and a control (sample stored for 0 days) [45] (see Table 2 for more details).

**Table 8:** Acids in pulses (expressed as percentages).

Acids	CAS	Origin (s)	Pea		Chickpea	Faba Bean	
			Dehulled	Proteins		Tannin	Storage
Acetic acid	64-19-7	AA ^2,3,4^			3.10–3.90	1.84–2.35	0.49–1.94
2-Methylbutanoic acid	116-53-0	N ^1^; AA ^1,2,4^		0.00–0.22		0.13	0.00–0.26
3-Methylbutanoic acid	503-74-2	AA ^2,3,4^	0.42	0.00–0.41		8.79–12.37	2.55–10.36
Pentanoic acid	109-52-4		0.48				
Hexanoic acid	142-62-1	FFA ^1^	0.73	0.00−Coelution	1.06–1.98	0.57–1.54	
2-Ethyl hexanoic acid	149-57-5		0.51	0.00–0.43			
Heptanoic acid	111-14-8		1.46				
Octanoic acid	124-07-2	N ^1^; FFA ^3^	1.54	0.00−Coelution			0.00–0.24
Nonanoic acid	112-05-0	FFA ^3^	1.92	0.00–1.40			0.00–0.58
Decanoic acid	334-48-5				0.00–0.89		
Palmitic acid	57-10-3				12.00–15.00		
Oleic acid	112-80-1				11.00–24.00		
Total			7.07	0.63–1.84	32.77–40.16	11.33–16.39	3.24–13.15

^1^ [42]; ^2^ [37]; ^3^ [45]; ^4^ [26]. AA, amino acids; N, naturally present (not considered as a contaminant); FFA, free fatty acids. The value “min–max” corresponds to the minimum and maximum percentages for each volatile compound identified. For peas, “Dehulled” corresponds to dehulled pea flour [42], and “Protein” refers to protein concentrate (dry process) [44] and protein isolate (wet process) [42]. “Chickpea” refers to 2 cultivars [43]. For faba beans, “Tannin” corresponds to a group of low-tannin cultivars and a group of high-tannin cultivars [37], and “Storage” corresponds to high-tannin cultivars that were stored under 3 different conditions (ambient, positive, and negative temperatures) for 60 days and a control (sample stored for 0 days) [45]. No acid was detected in black beans, pinto beans, red kidney beans, whole peas, and “location” faba beans (see Table 2 for more details).

**Table 9:** Esters in pulses (expressed as percentages).

Esters	CAS	Origin (s)	Black bean	Pinto Bean	Dark Red Kidney Bean	Pea			Chickpea	Faba Bean	
						Whole	Dehulled	Proteins		Tannin	Storage
Ethyl ethanoate	141-78-6	FFA ^3^				0.00–1.20					
2-Ethylhexyl ethanoate	103-09-3										0.00–0.79
Butyl ethanoate	123-86-4										0.00–0.25
Hexyl ethanoate	142-92-7	FFA ^2^									0.00–2.96
Ethyl cyanoacetate	105-56-6								0.00–0.39		
Decyl bromoacetate	5436-93-1		0.00–0.36	0.00–0.32	0.00–0.34						
1,2-Dihydro-2-naphtalenylacetate	-								0.80–1.06		
Ethyl propanoate	105-37-3						Coelution	0.00–17.19			
Octyl pivalate	-										0.00–0.76
Hexyl 2-methylbutanoate	10032-15-2										0.00–3.54
Ethyl butanoate	105-54-4						0.93	0.00–0.05			0.00–3.66
2-Ethylhexyl butanoate	25415-84-3										0.00–0.92
Methyl isovalerate	556-24-1	AA ^1^									0.00–2.11
Isoamyl isovalerate	659-70-1										0.00–0.51
Butyl hexanoate	626-82-4										0.00–7.48
2-Propenyl hexanoate	123-68-2						0.82				
Hexyl hexanoate	6378-65-0										0.00–3.33
Octyl hexanoate	4887-30-3							0.00–1.75			
Methyl salicylate	119-36-8									0.04–0.05	0.00–0.50
5-Isobutylnonane	62185-53-9								0.00–3.10		
4-Dodecanoyloxybutyl dodecanoate	624-07-7								0.00–0.77		
Isopropyl myristate	110-27-0								0.44–0.53		
3-Hydroxy-ethyl mandelate	-		0.28–0.42	0.34–0.45	0.00–0.42						
**Total**			0.42–0.66	0.45–0.66	0.00–0.77	0.00–1.20	1.75	1.75–17.25	1.50–5.59	0.04–0.05	0.00–24.15

^1^ [45]; ^2^ [35]; ^3^ [21]. FFA, free fatty acids; AA, amino acids. The value “min–max” corresponds to the minimum and maximum percentages for each volatile compound identified. “Black bean” refers to the 3 cultivars studied, and “Pinto bean” and “Dark red kidney bean” correspond to 2 cultivars for each [40]. For peas, “Whole” corresponds to 3 different harvest years of the cultivar Eclipse (2005, 2006, and 2007) [11], “Dehulled” corresponds dehulled pea flour [42], and “Protein” refers to protein concentrate (dry process) [44] and to protein isolate (wet process) [42]. “Chickpea” refers to 2 cultivars [43]. For faba beans, “Tannin” corresponds to a group of low-tannin cultivars and a group of high-tannin cultivars [37], and “Storage” corresponds to high-tannin cultivars that were stored under 3 different conditions (ambient, positive, and negative temperatures) during 60 days and a control (sample stored for 0 days) [45]. No ester was detected in “location” faba beans (see Table 2 for more details).

**Table 10:** Pyrazines in pulses (expressed as percentages).

Pyrazines	CAS	Origin (s)	Pea		
			Whole	Dehulled	Protein
2,3-Diethyl-5-methylpyrazine	18138-04-0		0.00–1.30		
2-Methyoxy-3-isopropyl(5or6)-methylpyrazine	32021-41-3	AA ^1^		2.62	0.00–0.13
2-Methoxy-3-isobutylpyrazine	24683-00-9	AA ^2^		Trace	
Total			0.00–1.30	2.62	0.00–0.13

^1^ [42]; ^2^ [34]. AA, amino acids. The value “min–max” corresponds to the minimum and maximum percentages for each volatile compound identified. For peas, “Whole” corresponds to 3 different harvest years of the cultivar Eclipse (2005, 2006, and 2007) [11], “Dehulled” corresponds to dehulled pea flour [42], and “Protein” refers to protein concentrate (dry process) [44] and protein isolate (wet process) [42]. No pyrazine was detected in black beans, pinto beans, dark red kidney beans, chickpeas, and faba beans (“tannin”, “location”, and “storage”) (see Table 2 for more details.).

**Table 11:** Terpenes in pulses (expressed as percentages).

Terpenes	CAS	Origin (s)	Black Bean	Pinto Bean	Dark Red Kidney Bean	Pea			Faba Bean	
						Whole	Dehulled	Proteins	Tannin	Storage
α-Pinene	80-56-8	CAR ^1,2^	1.37–2.29	2.11–3.92	0.75–1.07					
Δ3-Carene	13466-78-9	CAR ^1,2^	0.00–0.50	0.00–0.48		0.30–0.70				
Limonene	138-86-3	CAR ^1,2^	0.00–1.36	1.31–2.96			0.66	0.00–0.11	0.96	0.00–10.81
γ-Terpinene	99-85-4									0.00–0.79
Terpinolene	586-62-9									0.00–0.12
Thujospsene-I3	-									0.00–0.09
(E)-β-Ionone	79-77-6	CAR ^3^							0.11–0.12	
β-Myrcene	123-35-3	CAR ^2^								0.00–0.60
Geranylacetone	689-67-8		0.00–0.69		0.00–0.30					
Elemol	639-99-6						2.71			
α-Muurolol	19435-97-3						2.94			
t-Muurolol	19912-62-0						7.45			
α-Cadinol	481-34-5						Coelution			
β-Eudesmol	473-15-4						Trace			
β-Linalool	78-70-6									0.11–0.79
Menthol	1490-04-6								0.28–0.45	0.00–0.60
p-Menth-1,5-dien-8-ol	1686-20-0						Coelution			
Total			2.06–4.77	3.91–6.89	1.06–1.07	0.30–0.70	13.77	0.00–0.11	1.35–1.63	0.11–12.73

^1^ [11]; ^2^ [35]; ^3^ [25]. CAR, carotenoids. The value “min–max” corresponds to the minimum and maximum percentages for each volatile compound identified. “Black bean” refers to the 3 cultivars studied, and “Pinto bean” and “Dark red kidney bean” correspond to 2 cultivars for each [40]. For peas, “Whole” corresponds to 3 different harvest years of the cultivar Eclipse (2005, 2006, and 2007) [11], “Dehulled” corresponds to dehulled pea flour [42], and “Protein” refers to protein concentrate (dry process) [44] and protein isolate (wet process) [42]. For faba beans, “Tannin” corresponds to a group of low-tannin cultivars and a group of high-tannin cultivars [37], and “Storage” corresponds to high-tannin cultivars that were stored under 3 different conditions (ambient, positive, and negative temperatures) during 60 days and a control (sample stored for 0 days) [45]. No terpene was detected in chickpeas and “location” faba beans (see Table 2 for more details).

**Table 12:** Furans in pulses (expressed as percentages).

Furans	CAS	Origin (s)	Black Bean	Pinto Bean	Dark Red Kidney Bean	Pea			Faba Bean		
						Whole	Dehulled	Proteins	Tannin	Location	Storage
**2-Methylfuran**	534-22-5	FFA ^3^				0.00–1.80					
**2-Ethylfuran**	3208-16-0	FFA ^1,5^	0.00–0.64	0.62–0.74	0.00–0.58	0.00–3.90				0.29–0.29	
**2-Acetylfuran**	1192-62-7						Coelution	0.00–Trace			
**2-Propylfuran**	4229-91-8		0.53–0.71	0.49–0.61	0.75–0.85						
**2-Propionylfuran**	3194-15-8		0.44–0.66	0.00–0.78	0.53–0.69						
**2-Pentylfuran**	3777-69-3	FFA ^2,3,4,5^						0.00–0.91	0.60–0.78	1.72–1.91	0.61–1.43
**Total**			1.37–1.64	1.23–2.02	1.43–1.97	0.00–5.70		0.00–0.91	0.60–0.78	2.02–2.21	0.61–1.43

^1^ [41]; ^2^ [44]; ^3^ [45]; ^4^ [35]; ^5^ [14]. FFA, free fatty acids. The value “min–max” corresponds to the minimum and maximum percentages for each volatile compound identified. “Black bean” refers to the 3 cultivars studied, and “Pinto bean” and “Dark red kidney bean” correspond to 2 cultivars for each [40]. For peas, “Whole” corresponds to 3 different harvest years of the cultivar Eclipse (2005, 2006, and 2007) [11], “Dehulled” corresponds to dehulled pea flour [42], and “Protein” refers to protein concentrate (dry process) [44] and protein isolate (wet process) [42]. For faba beans, “Tannin” corresponds to a group of low-tannin cultivars and a group of high-tannin cultivars [37], “Location” corresponds to different low-tannin cultivars that were harvested at 2 different locations in Canada [41], and “Storage” corresponds to high-tannin cultivars that were stored under 3 different conditions (ambient, positive, and negative temperatures) for 60 days and a control (sample stored for 0 days) [45]. No furan was detected in chickpeas (see Table 2 for more details).

**Table 13:** Lactones in pulses (expressed as percentages).

Lactones	CAS	Origin (s)	Pea		Chickpea	Faba Bean	
			Dehulled	Proteins		Tannin	Storage
γ-Butyrolactone	96-48-0				0.00–0.85	0.32–0.63	0.22–1.08
3-Methylbutyrolactone	1676-49-8		0.45				
4-Methyl-4-vinylbutyrolactone	1073-11-6					0.08–0.09	
Pentolactone	599-04-2				2.20–2.30		
γ-Pentalactone	108-29-2		1.01				
δ-Caprolactone	823-22-3	N ^2^				0.24–0.26	0.00–0.82
γ-Caprolactone	695-06-7	N ^2^	10.11	0.00–Coelution		0.34–0.36	0.00–1.07
γ-Methyl-γ-caprolactone	2865-82-9		1.16	0.00–1.63			
4-Hydroxy-2-hexenoic acid lactone	2407-43-4			0.00–0.17			
γ-Octalactone	104-50-7	FFA ^1^	Trace	0.00-Trace			
γ-Nonalactone	104-61-0	FFA ^1^	1.31	0.00–0.33			
4-Hydroxy-2-noneic acid lactone	21963-26-8	FFA ^1^	0.72	0.00–1.04			
δ-Undecalactone	710-04-3	N ^2^			0.00–0.47	0.19–0.20	
Total			14.76	0.00–3.18	2.30–3.52	1.21–1.50	0.22–2.47

^1^ [42]; ^2^ [37]. FFA, free fatty acids; N, naturally present (not considered as a contaminant). The value “min–max” corresponds to the minimum and maximum percentages for each volatile compound identified. For peas, “Dehulled” corresponds to dehulled pea flour [42], and “Protein” refers to protein concentrate (dry process) [44] and protein isolate (wet process) [42]. “Chickpea” refers to 2 cultivars [43]. For faba beans, “Tannin” corresponds to a group of low-tannin cultivars and a group of high-tannin cultivars [37], and “Storage” corresponds to high-tannin cultivars that were stored under 3 different conditions (ambient, positive, and negative temperatures) during 60 days and a control (sample stored for 0 days) [45]. No lactone was detected in black beans, pinto beans, dark red kidney beans, whole peas, and “location” faba beans (see Table 2 for more details).

**Table 14:** Other volatiles in pulses (expressed as percentages).

Other Volatiles	CAS	Black Bean	Pinto Bean	Dark Red Kidney Bean	Pea			Chickpea	Faba Bean	
					Whole	Dehulled	Proteins		Tannin	Storage
Estragole	140-67-0									0.00–0.16
Benzothiazole	95-16-9					0.42	0.00–1.50	0.00–0.30		
4,5-Dimethylimidazole	2302-39-8							0.00–1.22		
Dimethyl sulphide	75-18-3				0.86–3.30					
Dimethyl disulphide	624-92-0				0.00–0.80					
3-Phenylindole	1504-16-1	0.00–0.84	0.49–1.18	0.72–0.73						
Methoxy-phenyl-oxime	-	0.43–0.88	0.00–0.62	0.00–0.72					3.39–3.60	
2,4-Dimethylbenzenamine	95-68-1	0.00–0.26	0.00–0.25	0.00–0.35						
2-(Trimethylsilylethynyl)pyridine	86521-05-3							0.00–0.40		
Total		0.72–1.72	1.18–1.37	0.72–1.80	1.26–3.40	0.42	0.00–1.50	0.00–1.92	3.39–3.60	0.00–0.16

The value “min–max” corresponds to the minimum and maximum percentages for each volatile compound identified. “Black bean” refers to the 3 cultivars studied, and “Pinto bean” and “Dark red kidney bean” correspond 2 cultivars for each [40]. For peas, “Whole” corresponds to 3 different harvest years of the cultivar Eclipse (2005, 2006, and 2007) [11], “Dehulled” corresponds to dehulled pea flour [42], and “Protein” refers to protein concentrate (dry process) [44] and protein isolate (wet process) [42]. “Chickpea” refers to 2 cultivars [43]. For faba beans, “Tannin” corresponds to a group of low-tannin cultivars and a group of high-tannin cultivars [37], and “Storage” corresponds to high-tannin cultivars that were stored under 3 different conditions (ambient, positive, and negative temperatures) during 60 days and a control (sample stored for 0 days) [45]. No other volatile was detected in “location” faba beans (see Table 2 for more details).

**Table 15:** Odour-active compounds identified in pea (*Pisum sativum* L.) and lupin (*Lupinus angustifolius* L.).

Chemical Classes	Volatiles	CAS	Origin (s)	Odour Threshold	Odour Descriptors	Pea		Lupin	
						Dehulled ^1^ (DM)	Protein Isolate ^1^ (DM)	Whole ^2^	Dehulled ^3^
**Aldehydes**	4-Ethylbenzaldehyde	53951-50-1		13 ppb ^5^	Sweet, honey, hay, dry vegetable (pea isolate)	(ND)	%D 83 (1.7 µg/g)		
	Phenylacetaldehyde	122-78-1	AA	4 ppb ^5^	Floral (pea isolate)	(ND)	%D 56 (2.1 µg/g)		
	Vanillin	121-33-5	N ^6^	53 µg/L (water) ^4^	Sweet, vanilla, caramel (peas, dehulled lupin)	%D 33 (0.1 µg/g)	%D 28 (0.3 µg/g)		FD 1024
	Ethylvanillin	121-32-4	N ^6^	100 ppb ^5^	Vanilla (dehulled lupin)				FD 256
	3-Methylthiopropanal	3268-49-3	AA	0.1 % (oil) ^5^	Potato (pea isolate)	(ND)	%D 94 (0.1 µg/g)		
	Hexanal	66-25-1	FFA	2.4 µg/L (water) ^4^	Grass, floral, fruity (dehulled pea)/Fresh grass (pea isolate)/Fresh green bean, green (whole lupin)	%D 50 (0.6 µg/g)	%D 61 (83.0 µg/g)	OI 10 (135 µg/g)	
	Heptanal	111-87-7	FFA		Green vegetable (pea isolate)	(ND)	%D 28 (16.2 µg/g)	(NO–4 µg/g)	
	Octanal	124-13-0	FFA	3.4 µg/L (water) ^4^	Floral, sweet, beany (whole lupin)			OI 26 (3 µg/g)	
	(E)-2-Octenal	2548-87-0	FFA	3–6 ppb ^5^	Pea, dry vegetable (pea isolate)/Fatty, green, cucumber (dehulled lupin)	(ND)	%D 94 (0.3 µg/g)	OI 98 (3 µg/g)	
	Nonanal	124-19-6	FFA	2.8 µg/L (water) ^4^	Solvent (dehulled pea)	%D 17 (0.9 µg/g)	(NO–9.5 µg/g)		
	(E)-2-Nonenal	18829-56-6	FFA	0.19 µg/L (water) ^4^	Beany, green (whole lupin)/Cardboard, fatty, green (dehulled lupin)			OI 49 (6 µg/g)	FD 256
	(Z)-2-Nonenal	60784-31-8	FFA ^3^	0.1 ppb ^5^	Cardboard (dehulled lupin)				FD 32
	(E,Z)-2,6-Nonadienal	557-48-2	FFA ^3^	45 µg/L (water) ^4^	Sweet, fatty (whole lupin)/Cucumber, green (dehulled lupin)			OI 10 (7 µg/g)	FD 256
	(E,E,Z)-2,4,6-Nonatrienal	100113-52-8	FFA ^3^		Nutty, oat-flake (dehulled lupin)				FD 256
	(E)-4,5-Epoxy-(E)-decenal	134454-31-2	FFA ^3^	0.19 µg/L (water) ^4^	Metallic (dehulled lupin)				FD 1024
	(E,E)-2,4-Decadienal	25152-84-5	FFA	0.038 µg/L (water) ^4^	Peanut, grilled meat (peas)	%D 94 (Trace)	%D 83 (Coelution)		
**Alcohols**	Pentanol	71-41-0	FFA	1.6–70 ppm ^5^	Grilled, dust (dehulled pea)	%D 28 (1.0 µg/g)	(NO–3.6 µg/g)		
	1-Penten-3-ol	616-25-1	FFA	400 ppb ^5^	Beany, green (whole lupin)	(NO–4.6 µg/g)	(NO–1.4 µg/g)	OI 29 (26 µg/g)	
	2-Methoxyphenol (guaiacol)	90-05-1		3–31 ppb ^5^	Sweet, phenolic (whole lupin)			OI 2 (4 µg/g)	
	2-Ethylhexanol	104-76-7			Floral (pea protein)		%D 56 (Coelution)		
	Octanol	117-87-5	FFA	42–480 ppb ^5^	Mushroom, vegetable (dehulled pea)	%D 83 (0.8 µg/g)	(NO–2.5 µg/g)		
	1-Octen-3-ol	3391-86-4	FFA	14 ppb ^5^	Mushroom, vegetable (peas)	%D 94 (1.7 µg/g)	%D 78 (4.2 µg/g)	(NO – 78 µg/g)	
	Nonanol	143-08-8	FFA	50–90 ppm ^5^	Pea, vegetable, silt, earth (dehulled pea)	%D 78 (0.8 µg/g)	(ND)		
**Ketones**	2-Acetyl-1-pyrroline	85213-22-5	AA ^3^	99,000 µg/L (water) ^4^	Popcorn (dehulled lupin)				FD 32
	Maltol	118-71-8	AA ^3^	35,000 µg/L (water) ^4^	Caramel (dehulled lupin)	(ND)	(NO – Coelution)		FD 256
	Sotolone	28664-35-9	AA ^6^	2.1 µg/kg (cellulose) ^4^	Spicy, savoury (dehulled lupin)				FD 256
	2,3-Butanedione	431-03-8	FFA ^4^	0.3–15 ppb ^5^	Sweet, caramel, vanilla (whole lupin)			OI 17 (79 µg/g)	
	4-Methyl-2-pentanone	108-10-1	FFA ^4^	240–540 ppb ^5^	Sweet, fruity, bubble gum (whole lupin)			OI 5 (0 µg/g)	
	2-Pentylfuran	3777-69-3	FFA	6 ppb ^5^	Oat, nutty (whole lupin)			OI 5 (172 µg/g)	
	1-Penten-3-one	1629-58-9	FFA ^4^	400 ppb ^5^	Soil, green, herbal (whole lupin)			OI 28 (32 µg/g)	
	1-Octen-3-one	4312-99-6	FFA ^3^	0.016 µg/L (water) ^5^	Mushroom, fungi (lupins)			OI 49 (2 µg/g)	FD 32
	3-Octen-2-one	1669-44-9	FFA	5–4 µg/L (water) ^4^	Soil, earthy (whole lupin)	(ND)	(NO–2.5 µg/g)	OI 13 (2 µg/g)	
	2,3-Octanedione	585-25-1			Mushroom, vegetable (peas)	%D 94 (Trace)	%D 78 (4.1 µg/g)		
	(Z)-Octan-1,5-dien-3-one	65213-86-7	FFA ^3^		Geranium, metallic (dehulled lupin)				FD 128
	(E,E)-3,5-Octadien-2-one	30086-02-3	FFA	0.15 ppm ^5^	Vegetable, hay, earth (pea isolate)	(ND)	%D 100 (24.4 µg/g)		
	Undecanone	112-12-9		7–82 ppb ^5^	Floral, fruity (peas)	%D 22 (Trace)	%D 39 (0.6 µg/g)		
	β-Ionone	79-77-6	CAR	3.5 µg/L (water) ^4^	Violet, flowery (dehulled lupin)				FD 512
**Acids**	Acetic acid	64-19-7	AA	99,000 µg/L (water) ^4^	Vinegar (dehulled lupin)				FD 32
	Phenylacetic acid	103-82-2	AA ^7^	6100 µg/L (water) ^4^	Beeswax, honey (dehulled lupin)				FD 256
	2-Methylbutanoic acid	116-53-0	N, AA	2200 µg/L (water) ^4^	Sweaty, fruity, cheese (dehulled lupin)	(ND)	(ND)		FD 2048
	3-Methylbutanoic acid	503-74-2	AA	490 µg/L (water) ^4^	Animal (dehulled pea)/Sweaty, fruity, cheese (dehulled lupin)	%D 22 (0.3 µg/g)	(ND)		FD 2048
	Pentanoic acid	109-52-4	AA ^3^	11,000 µg/L (water) ^4^	Sweaty, fruity, cheese (dehulled lupin)	(NO–0.3 µg/g)	(ND)		FD 32
	Hexanoic acid	142-62-1	FFA	93 ppb–10 ppm ^5^	Faeces, meat broth, sewer (pea isolate)	(NO–0.5 µg/g)	%D 83 (Coelution)		
	Heptanoic acid	111-14-8		640 ppb–10.4 ppm ^5^	Animal, vomit (pea isolate)	(NO–1.0 µg/g)	%D 22 (ND)		
**Lactones**	γ-Octalactone	104-50-7	FFA	6.5 g/L (water) ^4^	Floral, anise, mint (peas)/ Coconut, sweet (dehulled lupin)	%D 17 (Trace)	%D 94 (Trace)		FD 64
	γ-Nonalactone	104-61-0	FFA	9.7 µg/L (water) ^4^	Sweet, candies, coconut (dehulled pea)/Coconut, sweet (dehulled lupin)	%D 72 (0.9 µg/g)	(NO – 1.0 µg/g)		FD 256
	4-Hydroxy-2-noneic acid lactone	21963-26-8	FFA		Mint, strawberry, chlorophyll (peas)	%D 50 (0.5 µg/g)	%D 56 (3.2 µg/g)		
	γ-Decalactone	706-14-9	FFA ^3^	1–11 ppb ^5^	Peach, fruity (dehulled lupin)				FD 32
**Pyrazines**	3-Isobutyl-2-methoxypyrazine	24683-00-9	AA	62 µg/L (water) ^4^	Woody, fresh pea (whole lupin)/Green pepper, earthy (dehulled lupin)			OI 70 (9 µg/g)	FD 32
	2-Sec-Butyl-3-methoxypyrazine	24168-70-5	AA ^4^	1 ppb ^5^	Woody, green (whole lupin)			OI 54 (1 µg/g)	
	3-Isopropyl-2-methoxypyrazine	25773-40-4	AA ^4^	39 µg/L (water) ^4^	Soil, fresh green, woody (whole lupin)/Pea, green pepper (dehulled lupin)			OI 53 (0 µg/g)	FD 256
	5or6-Methyl-3-isopropyl-2-methoxypyrazine	32021-41-3	AA	3–7 ppb ^5^	Plastic, cardboard, hay (dehulled pea)/Hay, dry, vegetable (pea isolate)	%D 50 (1.8 µg/g)	%D 83 (0.4 µg/g)		
	3-Ethyl-2,5-dimethylpyrazine	13360-65-1			Potato, baked (whole lupin)			OI 65 (1 µg/g)	
**Others**	Benzothiazole	95-16-9	AA ^6^	80–450 ppb ^5^	Grilled meat (peas)	%D 39 (0.3 µg/g)	%D 39 (4.6 µg/g)		
	Dimethyl trisulfide	3658-80-8		0.0099 µg/L (water) ^4^	Faeces, meat broth, sewer (pea protein)/Meaty, metallic, sulphur (whole lupin)	(ND)	%D 83 (0.2 µg/g)	OI 14 (1 µg/g)	

^1^ [42]; ^2^ [54]; ^3^ [28]; ^4^ [55]; ^5^ [50]; ^6^ [31]; ^7^ [26]. DM, dry matter; FFA, free fatty acids; AA, amino acids; N, naturally present (not considered as a contaminant); CAR, carotenoids; ND, not detected; %D, percentage of detection; NO, detected but not odorant; OI, odour intensity; FD, factor dilution.

## Data Availability

Not applicable.
